# First Principles Rovibronic Absorption Spectra of HF Molecule

**DOI:** 10.1002/jcc.70317

**Published:** 2026-02-24

**Authors:** Nariman Abu El Kher, Maha Shibli, Mahmoud Korek, Sergei N. Yurchenko, Jonathan Tennyson, Nayla El‐Kork

**Affiliations:** ^1^ Department of Physics Khalifa University Abu Dhabi UAE; ^2^ Department of Physics Beirut Arab University Beirut Lebanon; ^3^ Department of Physics and Astronomy University College London London UK

**Keywords:** ab initio calculations, line lists, radiative lifetime, rovibronic spectrum, spectroscopic model

## Abstract

An ab initio study on the rovibronic spectroscopy of the hydrogen fluoride HF molecule is reported based on using high‐level electronic structure computations and accurate calculations of the Schrödinger equation for the nuclear motion. A combination of empirical and ab initio methods is used to generate a spectroscopic model and a corresponding line list of the HF molecule. The line lists cover the 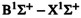 and 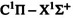 band systems. Rovibronic absorption spectra are simulated in the form of temperature‐dependent cross sections for these bands. Comparisons between our simulated absorption cross section and the available experimental ones show overall consistency. Calculated radiative lifetimes ($\tau_{v^{\prime }}$) for the 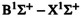 and 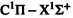 systems are compared to previous results. Given the scarcity of published rovibronic experimental data on this molecule, the line lists can be used for spectroscopic modeling of HF in astrophysical media, including interstellar space, planetary and exoplanetary atmospheres.

## Graphical abstract

Hydrogen fluoride (HF) is astrophysically important, as it has been detected in sunspots, Venus, red giants, and the interstellar medium, serving as a probe of exoplanet atmospheres. This article presents an ab *initio* investigation of HF rovibronic spectroscopy, combining high‐level electronic‐structure data with nuclear‐motion solutions and refinement to produce validated line lists. Temperature‐dependent absorption cross sections are simulated and agree with measurements. Radiative lifetimes are calculated, enabling interpretation and modeling in astrophysical environments.

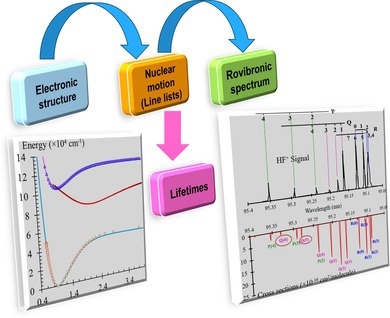

## 1 Introduction

Hydrogen fluoride has been detected in astronomical environments, often in significant amounts. It is present in sunspots [1, 2], the atmosphere of Venus [3], red giants [4], and the interstellar medium [5]. Hydrogen fluoride is expected to be the primary form of fluorine gas found in most interstellar clouds, containing most of the fluorine atoms present in the gas phase under a wide range of cloud conditions [5]. Neufeld et al. [6] proposed a chemical model explaining how fluorine‐containing molecules behave in diffuse and dark clouds exposed to ultraviolet radiation. Their model predicts high levels of hydrogen fluoride in such clouds. The Heterodyne Instrument for the Far‐Infrared instrument [7] on board the *Herschel Space Observatory* [8] facilitated the study of HF ground‐state rotational transitions in interstellar space. Numerous observations of HF in diffuse clouds along the lines of sight to submillimeter continuum sources, including the high mass star formation regions W31C [9], W49N, W51 [10], and Sgr B2(M) [11], have been documented. These observations demonstrate that HF serves as an excellent tracer of ${\text{H}}_{2}$ in diffuse clouds, compared to the commonly used CO, consistent with the predictions of the model, suggesting that the abundance of HF can exceed that of CO in such media [6]. HF emissions are also important in terrestrial applications such as lithium‐ion battery fires [12]. The analysis and understanding of such observations require a thorough knowledge of the spectral signatures of the HF molecule.

Experimental spectroscopic investigation of hydrogen fluoride in the ultraviolet range dates back to the mid‐20th century. In 1951, Safary et al. [13] reported the observation of a continuous absorption spectrum, which was assigned to a low‐lying repulsive state of the molecule. In 1959, Johns and Barrow [14] analyzed an emission band system that arised from a 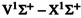 electronic transition, where 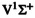 denoted the lowest stable excited singlet state. Subsequent studies [15, 16, 17] reported observation of the vibration‐rotation bands of the hydrogen fluoride molecule in its 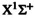 ground state. Di Lonardo and Douglas [18, 19] published a detailed report on the absorption and emission bands associated with 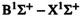 band system. In addition, a more detailed analysis of the absorption spectra of the bands associated with the lowest excited states at high dispersion in the vacuum ultraviolet region was conducted by Douglas and Greening [20] in 1979; however, they could not assign the region associated with the molecule's Rydberg states. Assignment of HF's Rydberg structure was proposed by Hitchcock et al. [21, 22], who performed detailed experimental and theoretical studies of the electron energy loss spectrum between 7 and 46 eV. Rotationally‐resolved spectra of the HF 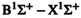 and 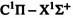 band systems were observed by Tashiro et al. [23] by 1 XUV +1 UV Resonance‐Enhanced Multiphoton Ionization (REMPI). The spectral wavelength for these band systems was extended from 93.8 to 99.0 nm. Despite the sharp and well‐resolved spectral lines for transitions within its low‐lying electronic states, there has been very little progress in analyzing HF's absorption spectra in the vacuum ultraviolet region experimentally. Among several reasons, the highly corrosive nature of the vapor on the surfaces of optical components used in HF experiments constitutes a barrier to such investigations [24].

Hydrogen fluoride is one of the well‐known ten‐electron systems, together with water, ammonia, and methane [25]. These species constitute a widely used test set for the development and validation of quantum chemical methods [25]. As a simple heteronuclear molecule, HF serves as a benchmark system for electronic structure characterization, and many previous comprehensive studies [26, 27, 28, 29, 30, 31, 32, 33, 34, 35, 36, 37, 38] are available.

Huber and Herzberg [39] provide experimental spectroscopic constants for three low‐lying singlet electronic states (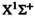, 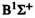, and 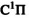) of the HF molecule. No experiments have been carried out on the triplet states of HF molecules; however, in 1982, Bettendorff et al. [29] calculated their potential energy curves (PECs) at the MCSCF level and deduced their adiabatic electronic energy values ($T_{e}$) relative to the ground state. Subsequently, in 1997, Feller and Peterson [33] determined the spectroscopic constants for the singlet and triplet states. Later on in 2014, ab initio studies on HF were performed by Huang et al. [36] using a multireference configuration interaction (MRCI) approach for various states. The most recent high‐level ab initio study was carried out by Liu et al. [37] using a multi‐reference configuration interaction method plus Davidson correction (MRCI+Q).

Recently, Pezzella et al. [40, 41] and Qin et al. [42] computed temperature‐dependent photodissociation cross sections for HF, focusing on the first three electronic excited states (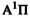, 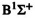, and 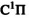), which relied mainly on the ab initio PECs and TDMs reported by Liu et al. [37]. Even though these curves are suitable for photodissociation investigations, the PECs and TDMs used are insufficiently accurate for high‐resolution line list calculations. For example, when adopted for the analysis of the B‐X vibronic bands of HF molecule, these PECs and TDMs result in a systematic shift of the calculated spectra with respect to experiment, amounting to approximately 1500 ${\text{cm}}^{-1}$. Motivated by this discrepancy, the present work focuses on improving and refining the current electronic structure curves of HF molecule, to achieve an accurate spectroscopic model and a quantitatively consistent spectral analysis of hydrogen fluoride.

To this end, first‐principles investigations of the electronic structure of the hydrogen fluoride molecule are carried out, leading to fitted and refined PECs for the B and C states. The resulting optimized curves lead to a comprehensive rovibronic line list for the 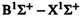 and 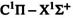 transitions, and theoretically generated rovibronic photo‐absorption spectra which now compare well against available experimental measurements. The calculations are performed using a combination of empirical and ab initio PECs and electronic angular momentum curves (EAMCs), in conjunction with high‐level ab initio permanent dipole moment curves (PDMCs) and transition dipole moment curves (TDMCs). In addition, singlet and triplet states, associated with four dissociation limits, are computed at the MRCI + Q level. To facilitate the assessment of our results, the spectroscopic constants ($T_{\text{e}}$, $R_{\text{e}}$, $\omega_{\text{e}}$, $\beta_{\text{e}}$, $\omega_{\text{e}}x_{\text{e}}$), the dipole moment $\mu_{\text{e}}$, and the dissociation energies $D_{\text{e}}$ are calculated and compared with literature values.

The paper is structured as follows. Section 2 describes the method followed for computing the PECs of HF molecule and selected coupling TDMCs and EAMCs among states of interest. Rovibronic line list calculations are also presented in the same section. Section 3 presents the ab initio and line lists results, and Section 4 outlines an analysis of the obtained spectra and corresponding radiative lifetimes in terms of comparisons with the literature. Finally, conclusions are discussed in Section 5.

## 2 Spectroscopic Model Calculation Methodology

### 2.1 Electronic Structure Computations

The ab initio calculations were conducted using the MOLPRO 2022.1.2 [43] software package and the GABEDIT graphical user interface [44]. The MOLPRO code is widely used in quantum chemistry because it can describe electron correlation very accurately, which is important for obtaining reliable results on molecular systems [43]. The electronic structure of the HF molecule was calculated using a state‐averaged complete active space self‐consistent field (CASSCF) approach, followed by an MRCI+Q calculation. The calculations were based on the $C_{2v}$ finite point group, a subset of the $C_{\infty v}$ infinite symmetry group, using the irreducible representations ($A_{1}$, $A_{2}$, $B_{1}$, and $B_{2}$) in $C_{2v}$ symmetry. The $A_{1}$ irreducible representation results in $\Sigma^{+}$ states and Δ states, $A_{2}$ gives the $\Sigma^{-}$ states and the other component of Δ states, and $B_{1}$ yields the Π states. A well‐known challenge of this methodology is producing smooth curves, such as PECs and TDMCs, since orbital swapping with changing bond length often introduces artificial jumps or oscillations in the results [45].

In this work, the augmented correlation‐consistent polarized valence five‐zeta basis set (aug‐cc‐pV5Z) [46] was selected for the hydrogen atom (H), and the fluorine atom (F) was treated using the augmented correlation‐consistent polarized valence quadruple‐zeta with relativistic‐contracted Douglas–Kroll basis set (aug‐cc‐pVQZ‐DK) [47]. Only the spd functions were adopted, while the electrons in d‐type atomic orbitals were excluded from the calculation to reduce orbital swapping and obtain smoother PECs. In fact, tests performed with the full spdfg functions and with correlated d‐type orbitals have increased the error in the estimation of the spectroscopic constants, particularly for excited states. The corresponding data are provided in Table S1 in the Supporting Information. Thirteen molecular orbitals $7a_{1}$, $0a_{2}$ $3b_{1}$, and $3b_{2}$, were selected as the active space in the $C_{2v}$ point group, arising from 7σ (H: 1s, 2s, 3s, $2p_{0}$); (F: $2p_{0}$, 3s, $3p_{0}$), 3π (H: $2p_{\pm 1}$; F: $2p_{\pm 1}$, $3p_{\pm 1}$). The fluorine 1s and 2s orbitals were treated as the core and kept doubly occupied in all electronic configurations. A CASSCF calculation was performed with six valence electrons from HF distributed over the thirteen active orbitals, defining a CAS (6,13) active space. The four inner shell electrons were assigned to two closed‐shell orbitals of $a_{1}$ symmetry, corresponding to the 1σ and 2σ molecular orbitals in the HF molecule.

The selection of basis sets and active spaces in this study was not arbitrary but guided by systematic testing. The comparison was based on the calculated energy levels of several molecular states of HF at the lowest dissociation limits. As an illustration, configuration 1 refers to a validatory comparative calculation carried out using a given basis set and active space, whereas configuration 2 denotes the main methodology adopted in this work. In configuration 1, the H atom was described with the cc‐pV5Z basis set [46] without diffuse functions (in contrast to configuration 2), whereas the F atom was treated with the same basis set used in configuration 2. The active space in configuration 1 comprised the $7a_{1}$, $3b_{1}$, $3b_{2}$, and $1a_{2}$ orbitals, which included d‐type atomic orbitals. In configuration 2, the adopted active space consisted of $7a_{1}$, $3b_{1}$, $3b_{2}$, and $0a_{2}$, thereby excluding the d‐type orbitals.

Table 1 shows the energy levels for different molecular states of the HF molecule at the lowest dissociation limits, using the two configurations. A comparison of these results with experimental values [48] is also given. The calculated energy separation of the dissociation limit H(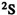) + F(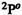) for configuration 1 is 92,755.24 ${\text{cm}}^{-1}$, revealing a deviation of 10,496.29 ${\text{cm}}^{-1}$ (12.8%) from the experimental value (82,258.95 ${\text{cm}}^{-1}$). In contrast, the calculated energy separation (82,376.16 ${\text{cm}}^{-1}$) of the first dissociation limit obtained with configuration 2 tends to converge and closely match the experimental value (82258.95 ${\mathrm{cm}}^{-1}$), where the difference is only 117.21 ${\text{cm}}^{-1}$ (0.1%). Additionally, when employing configuration 1, the higher‐lying PECs lack smoothness, particularly in the asymptotic region. Such undesirable broken features are absent in configuration 2. These improvements result from the addition of the diffuse function to the H atom, the increase in the function flexibility (by using a higher‐order basis set) for the F atom, and the exclusion of electrons in d‐type atomic orbitals from the calculation in configuration 2. Such enhancements emphasize the sensitivity of excited‐state PECs to the choice of computational parameters. Therefore, configuration 2 is found to be more suitable for computing the electronic structure of the HF molecule.

**TABLE 1:** Lowest dissociation limits and associated molecular states the HF molecule calculated using the MRCI+Q method for configurations 1 and 2.

Atomic states	Molecular states	Energy separation (in 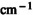 )				
H+F	HF	$E_{\exp t.}$ ^a^	$E_{theo.1}$ ^b^	% rel. error	$E_{theo.2}$ ^b^	% rel. error
H(1s, 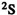 ) + F($2s^{2}2p^{5}$, 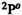 )	$X^{1}\Sigma^{+}$, ${\left(1\right)}^{1}\Pi$, ${\left(1\right)}^{3}\Sigma^{+}$, ${\left(1\right)}^{3}\Pi$	0.00	0.00	0.0	0.0	0.0
H (2s, 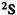 ) + $F(2s^{2}2p^{5}$, 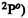	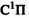 , ${\left(3\right)}^{1}\Sigma^{+}$, ${\left(2\right)}^{3}\Pi$, ${\left(2\right)}^{3}\Sigma^{+}$	82,258.95	92,755.24	12.8	82,376.16	0.1
H (2p, 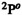 ) + F($2s^{2}2p^{5}$, 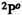 )	${\left(4\right)}^{1}\Sigma^{+}$, ${\left(1\right)}^{1}\Sigma^{-}$, ${\left(3\right)}^{1}\Pi$, ${\left(1\right)}^{1}\Delta$,	82,259.16	^c^	—	85,130.48	3.5
	${\left(3\right)}^{3}\Sigma^{+}$, ${\left(1\right)}^{3}\Sigma^{-}$, ${\left(3\right)}^{3}\Pi$, ${\left(1\right)}^{3}\Delta$					

^a^ J‐weighted average values, from NIST [48].

^b^ Present work ($E_{theo.1}$ corresponds to configuration 1 and $E_{theo.2}$ corresponds to configuration 2).

^c^ Region of fluctuations, see text in Section 2.

### 2.2 Nuclear Motion Computations

The program Duo, developed by Yurchenko et al. [49], is a highly flexible code that provides variational solutions to the nuclear motion problem for diatomic molecules. It can compute rotational, rovibrational, and rovibronic line lists based on electronic structure calculations or semi‐empirical data. To achieve this, high‐level ab initio PECs in conjunction with TDMCs, and, if relevant, spin‐orbit coupling curves (SOCCs), and EAMCs are used in the Duo input files. Refining these curves using available experimental data is desirable to obtain highly precise results. Duo also offers additional features, such as interpolating and extrapolating data to generate line lists that include line positions, intensities, and Einstein coefficients. The primary approach employed by Duo to solve the radial equation is the Sinc Discrete Variable Representation (DVR) method, known for its faster convergence of calculated energies and wave functions relative to the number of grid points, $N_{p}$.

In this work, Duo calculations were performed using a grid‐based sinc DVR basis. A grid of 2501 points covering a range from 0.02 to 25 Å was used for the 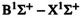 transition, and 501 points spanning 0.02 to 5 Å were used for the 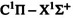 transition. Such ranges ensured that all vibrational states below the dissociation limits were included for each state. The final vibrational basis set consists of the 50, 60, and 60 lowest vibrational eigenfunctions for the 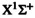, 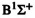, and 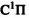 states, respectively. Duo distinguishes between bound and unbound states, where the latter appear above the dissociation limit. In this work, the unbound states have been identified using the probability density method in Duo. Several trials were conducted to obtain the optimal cutoff threshold of the wavefunction density at the grid boundary for the states involved in the studied transition.

## 3 Results and Discussion

### 3.1 Ab initio Results

**FIGURE 1:**
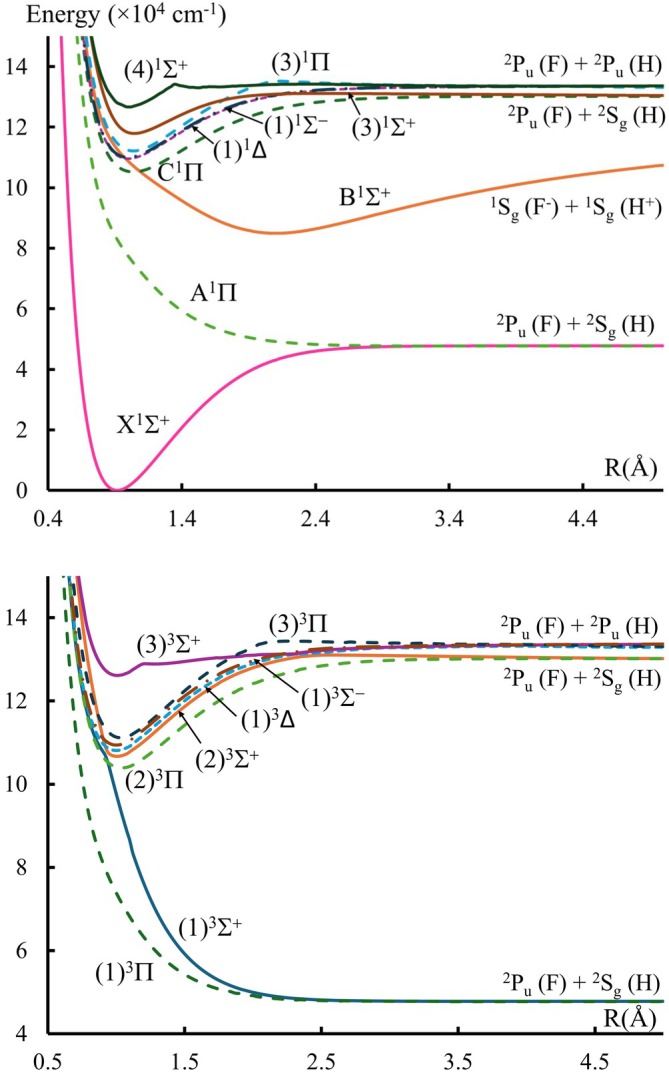
Potential energy curves of the singlet (upper panel) and triplet (lower panel) states of the HF molecule.

The PECs of seventeen spin‐orbit coupling free electronic states in the Λ−S representation in both singlet and triplet multiplicities have been investigated and displayed as a function of internuclear separation in Figure 1. These states correlate with four asymptotic dissociation limits; the origin of each limit (arising from a combination of atomic orbitals of H and F atoms) is represented in the figures for clarity. The lowest dissociation limits displayed in Table 1 agree with the experimental data within a percentage relative difference of 0.1% and 3.5% for the second and third dissociation limits, respectively.

The dissociation limit of the 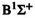 excited state is not included in Figure 1 and Table 1 because of the nature of its bonding at large internuclear distance, which is almost entirely ionic. In fact, the molecule dissociates there into the ionic fragments 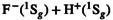. This state was assigned previously as the 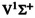
state by Mulliken [50], who identified its nature. Subsequently, Johns and Barrow [14] and Douglas and Greening [20] also designated the same state as an ionic state, and called it the 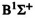 state. Accordingly, and to be consistent with the latest notation, the given state will here be called the upper 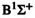 state. A more comprehensive figure of this state is provided by Figure 8 below.

The spectroscopic constants that include the transition energy with respect to the minimum of the ground state $T_{\text{e}}$, the equilibrium bond length $R_{\text{e}}$, the harmonic frequency $\omega_{\text{e}}$, the anharmonicity constant $\omega_{\text{e}}x_{\text{e}}$, the rotational constant $\beta_{\text{e}}$, the dipole moment $\mu_{\text{e}}$, and the dissociation energy $D_{\text{e}}$ of the bound electronic states of the HF molecule are calculated and compared with the literature values. The constants for the 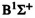 and 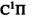 states, which are of primary interest in the present line‐list analysis, are reported in Table 2, whereas those for the remaining bound electronic states are provided in Table TS2 in the Supporting Information.

**TABLE 2:** Spectroscopic constants of ab initio Λ−S electronic states of the HF molecule. Values that include subscripts indicate the last digit's uncertainty, as described by Huber and Herzberg [39]; actual uncertainties may exceed ±10 units of the last digit. Numbers in parentheses (Obs.−Calc.) are based on the only available experimental data [39], given in italics.

States	Method	Ref.	$R_{\text{e}}$	$T_{\text{e}}$	$\omega_{\text{e}}$	$\omega_{\text{e}}x_{\text{e}}$	$B_{\text{e}}$	$D_{\text{e}}$	$\mu_{\text{e}}$
			(Å)	$\left({\text{cm}}^{-1}\right)$	$\left({\text{cm}}^{-1}\right)$	$\left({\text{cm}}^{-1}\right)$	$\left({\text{cm}}^{-1}\right)$	(eV)	$\left(\text{e}a_{0}\right)$
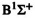	*Expt.*	[39]	*2.0908_6_*	*84,776.65*	*1159.18*	*18.005*	*4.0291*		
	MRCI+Q	This work	2.099 (−0.0082)	84,904.7 (−128.05)	1257.5 (−98.32)	151.0 (−133)	4.00 (0.0291)		2.87
	CASSCF/MRCI+Q	This work	2.091 (−0.0002)	85,755 (978.35)	1160.2 (−1.02)	17.73 (0.275)	4.03 (−0.0009)		
	(two‐states averaged)								
	IVO‐CASCI	[28]	2.032 (0.0588)		1042.8 (116.38)				
	$H_{3^{rd}}^{v}$	[28]	2.032 (0.0588)		1137.9 (21.28)				
	MRDCI	[29]	2.152 (−0.0612)	85,494 (−717.35)	1131 (28.18)		3.81 (0.2191)		
	iCAS‐CI	[33]	2.060 (0.0308)	86,301 (−1524.35)	1174 (−14.82)	−83.5 (101.505)	4.15 (−0.1209)		
	MRCI	[36]	2.094 (−0.0032)	84,967 (−190.35)	1139 (20.18)		4.02 (0.0091)		
	MRCI+Q	[37]	2.015 (0.0758)	83,299 (1477.65)	1193 (−33.82)	14.16 (3.845)	4.11 (−0.0809)	5.917	
	CASSCF/MRCI+Q	[37]	2.087 (0.0038)	85,033 (−256.35)	1161 (−1.82)		4.05 (−0.0209)		
	(Two‐states averaged)								
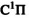	*Expt.*	[39]	*1.04_9_*	*105,820*	*2636*		*16.0*		
	MRCI+Q	This work	1.038 (0.002)	105,211.4 (608.6)	2639.4 (−3.4)	51.78	16.32 (−0.32)	3.091	1.74
	iCAS‐CI	[33]	1.029 (0.011)	104,851 (969)	2816 (−180)		16.6 (−0.6)		
	MRCI+Q	[37]	1.027 (0.013)	106,264 (−444)	2862 (−226)	73.19	16.65 (−0.65)	3.188	

Two different state average calculation methods were considered for the first excited state 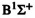, namely:

i. the state‐averaged CASSCF/MRCI+Q. In this method, the CASSCF orbitals are optimized by averaging over a selected set of electronic states. In this work, the averaging was performed over all the considered electronic states.
ii. The two states‐averaged CASSCF/MRCI+Q. In this method, the orbitals are optimized by averaging over two electronic states simultaneously, in this case the ground 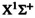 and the first excited 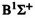 states.

Trying different methods aims at a better understanding of the significance of state mixing in MRCI calculations and their effect on the shape of the calculated 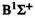 PEC. As presented in Table 2, the spectroscopic constants of the 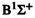 state obtained from the two states‐averaged CASSCF/MRCI+Q method exhibit close agreement with experimental data [39], with deviations of $\delta R_{\text{e}}=0.0002$ Å, $\delta \omega_{\text{e}}=1.02\;{\text{cm}}^{-1}$, and $\delta B_{\text{e}}=0.0009\;{\text{cm}}^{-1}$. In particular, the error in $R_{e}$ is well within the experimental uncertainty of $R_{e}=2.0908_{6}\;\text{Å}\;(\approx \pm 0.0006\;\text{Å})$. In contrast, the state‐averaged CASSCF/MRCI+Q method gives much larger discrepancies: $\delta R_{\text{e}}=0.0082$ Å, $\delta \omega_{\text{e}}=128.05\;{\text{cm}}^{-1}$, and $\delta B_{\text{e}}=0.0291\;{\text{cm}}^{-1}$ with the same data. Our spectroscopic constants for the 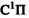 state agree well with the experimental ones given by Huber and Herzberg [39], with differences for $T_{\text{e}}$, $R_{\text{e}}$, $\omega_{\text{e}}$, and $B_{\text{e}}$ that are respectively equal to $608.6\;{\text{cm}}^{-1}$ (0.6%), 0.002 Å (0.2%), $3.4\;{\text{cm}}^{-1}$ (0.1%), and $0.32\;{\text{cm}}^{-1}$ (2.0%). Notably, the error in $R_{e}$ is well within the experimental uncertainty of $R_{e}=1.04_{9}\;\text{Å}\;(\approx \pm 0.09\;\text{Å})$, so our calculated value of $R_{e}=1.038\;\text{Å}$ is consistent with the experiment. For higher excited states, the spectroscopic constants obtained in this work compare generally well with the theoretical data in the literature for both the singlet and triplet states.

**FIGURE 2:**
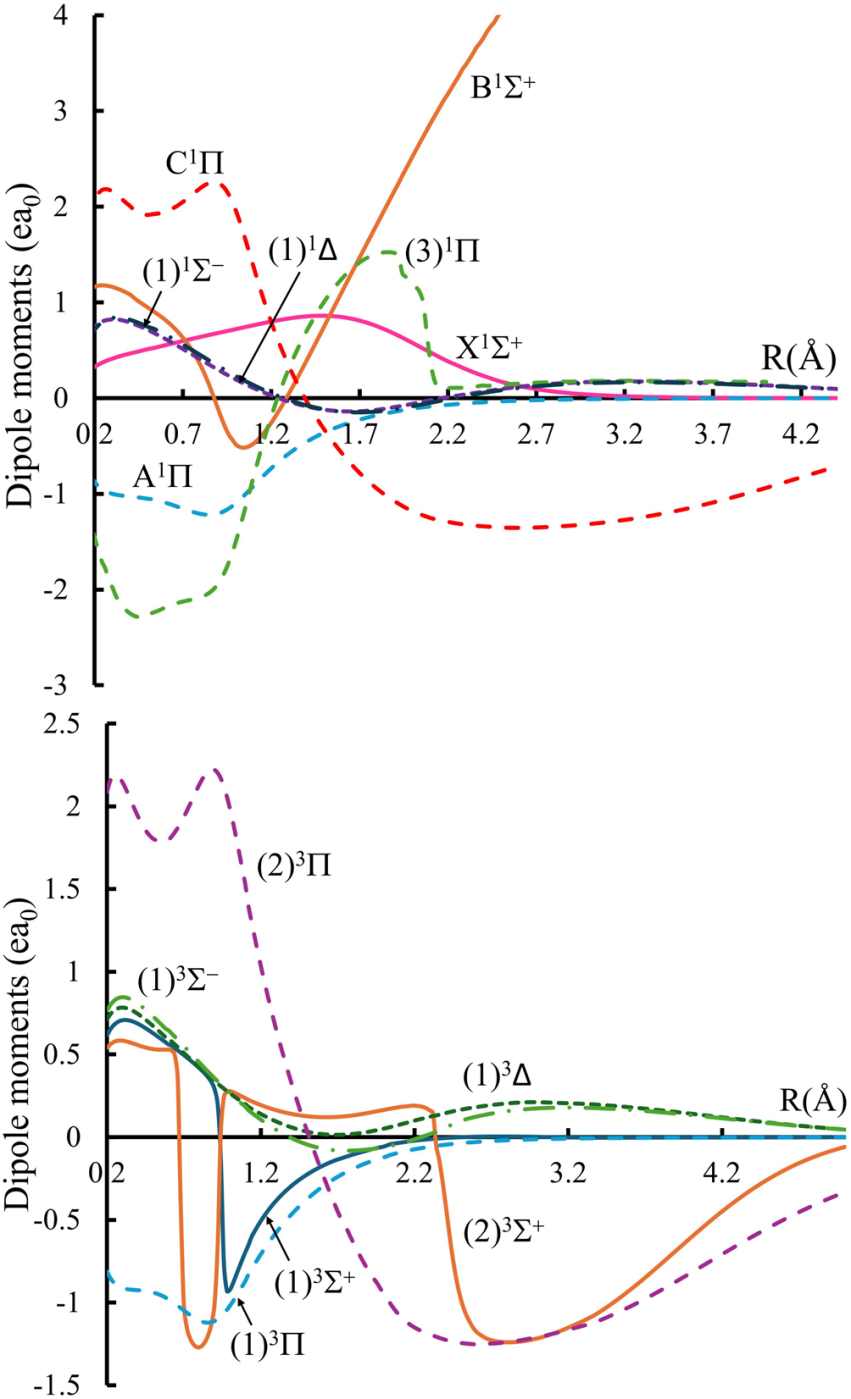
Calculated permanent dipole moment curves (PDMCs) of the singlet (upper panel) and triplet (lower panel) states of the HF molecule.

The PDMCs of the states of the HF considered here are plotted in Figure 2. The geometry of the HF system in the calculation of the DMCs is such that F is taken at the origin. As a result, negative dipole moment values indicate a charge displacement from F to H, reflecting a reversed polarity, represented as ${\text{H}}^{\delta -}{\text{F}}^{\delta +}$.

The calculated dipole moment for the ground state 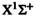 at the equilibrium position $R_{\text{e}}$ is positive with value $\mu_{\text{e}}=0.\;68\;\text{e}a_{0}$, indicating that the polarity of the HF molecule at this geometry is ${\text{H}}^{\delta +}{\text{F}}^{\delta -}$. Comparing this value with the experimental result of 0.707 $\text{e}a_{0}$ [26] and the previous theoretical value 0.68 $\text{e}a_{0}$ [28] shows acceptable agreement. Figure 2 shows that as the internuclear distance increases, the dipole moments of several electronic states undergo a sign change. Furthermore, the dipole moments of the ${\left(1\right)}^{3}\Sigma^{+}$ and ${\left(2\right)}^{3}\Sigma^{+}$ triplet states undergo abrupt gradient change at R=0.94 Å due to the occurrence of avoided crossing between their PECs, as shown in Figure 1.

**FIGURE 3:**
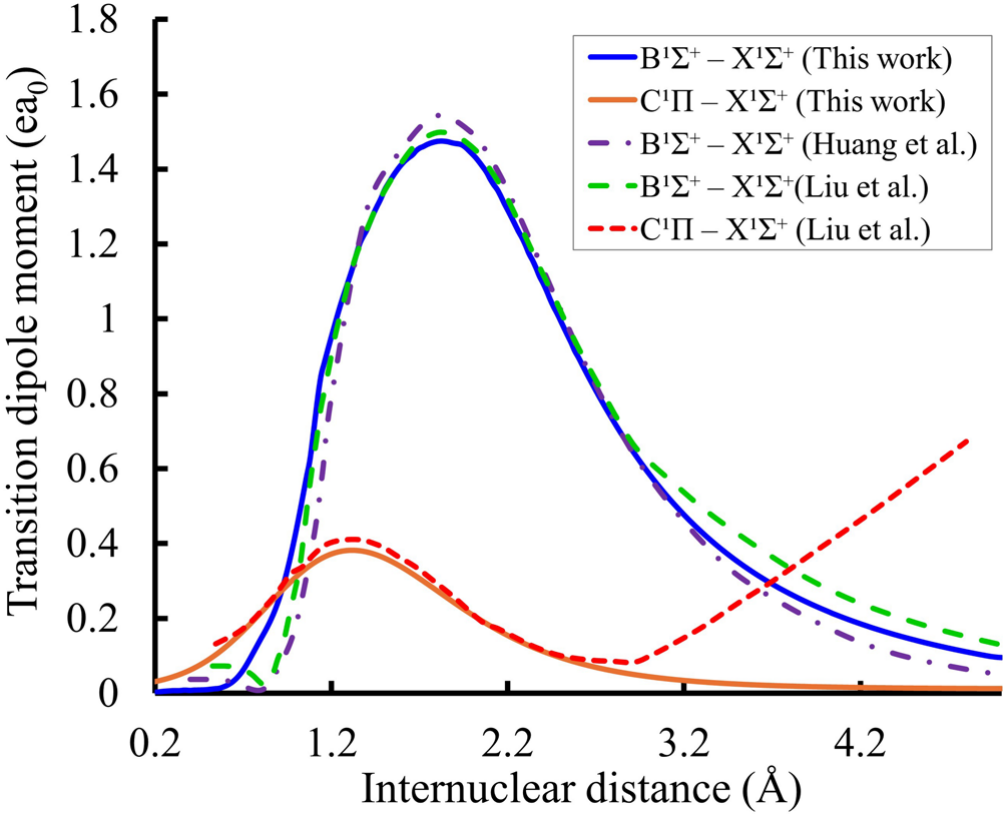
HF transition dipole moment curves compared with those calculated by Huang et al. [36] for 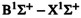 and Liu et al. [37] for the 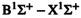 and 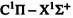.

The 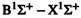 and 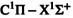 TDMCs of the HF molecules were computed as a function of the internuclear distance based on the state‐averaged MRCI+Q calculation. Our calculated TDMCs are compared in Figure 3 with those calculated by Huang et al. [36], who provided data for 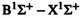 transition, and Liu et al. [37], who reported both 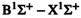 and 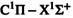 transitions. We smoothed our dipole moment curve for the 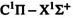 transition by fitting it with a Pearson IV function using the Origin program, where the residual standard deviation is $\sigma_{r}=0.00518$ $\text{e}a_{0}$. The initial and smoothed TDMCs are shown in Figure S1 of the Supporting Information.

The difference in the asymptotic behavior of our calculated 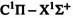 TDM and that reported by Liu et al. [37] can be attributed to the dissociation limits of the 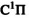 state in each calculation. In our case, it appears that the C state correlates at large R with the H(2s) + F limit, for which the H(2s) → H(1s) transition is electric dipole forbidden, resulting in the transition dipole moment approaching zero. In contrast, the 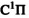 state of Liu et al. [37] correlates with the limit H(2p) + F, where the transition H(2p) → H(1s) is allowed, giving rise to a non‐zero TDM at large R.

**FIGURE 4:**
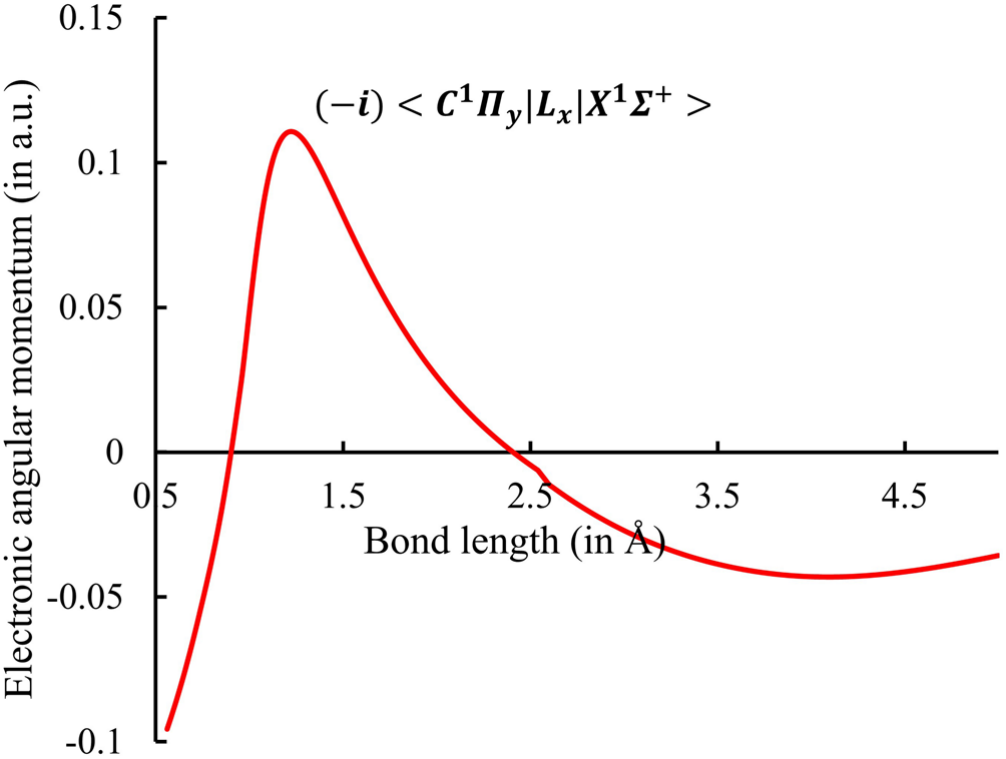
Electronic angular momentum curve for HF 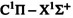.

The angular momentum coupling between the ground state 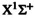 and the low‐lying electronically excited states considered in this work is analyzed by evaluating the non‐zero matrix elements of the electronic angular momentum operators $L_{x}$, $L_{y}$, and $L_{z}$, subject to the relevant selection rules [51, 52]. For the $L_{x}$ and $L_{y}$ components, non‐zero couplings are allowed under the selection rules ΔΛ=±1, ΔΣ=0, and ΔS=0. Consequently, the allowed angular momentum coupling curve for the 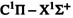 transition is calculated and illustrated in Figure 4, whereas such coupling is forbidden for the 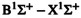 transition. The computed EAMC of 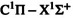 transition curve has been partially interpolated due to the presence of discontinuities arising from missing data points at certain geometries. To our knowledge, this curve is calculated for the first time, and as a consequence, comparison with previous results is not possible.

The vibrational energy levels of the HF molecule were also computed using the canonical functions approach [53, 54, 55] and the cubic spline interpolation of the PEC between every two consecutive points, for better characterization of the spectroscopic model and the obtained corresponding curves. The ro‐vibrational constants, such as the vibrational energy $E_{\nu }$, the rotational constant $B_{\nu }$, the centrifugal distortion constant $D_{\nu }$, and the abscissas of the turning points $R_{\min}$ and $R_{\max}$, have been calculated for the lowest vibrational states for both bound singlet and triplet electronic states based on the state‐averaged CASSCF/MRCI+Q calculations. The ro‐vibrational constants of the states 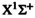, 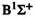, 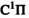, are presented in Table S3 of the Supporting Information, while those for the higher excited states are given in Table S4 of the Supporting Information. While taking into account the experimental uncertainties quoted in Table S3 of the Supporting Information, a comparison reveals preliminary agreement between our results, obtained prior to any refinement or fitting procedures, and available experimental data [19, 20, 23, 56, 57]. These studies mainly focus on the ground state and the two low‐lying excited states, 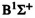 and 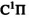.

### 3.2 Duo Results

When calculating rovibronic transitions, several trials are usually tested before adopting the electronic structure and coupling curves that would best represent experimental data. In such cases [58, 59, 60, 61], empirical curves can be used to produce reliable results. These are usually obtained by fitting/shifting the corresponding analytical curves to obtain energy terms that are close to the experimental ones. The ground 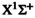 state energy levels obtained directly from this work's ab initio results were not accurate as judged by their discrepancy with experimental term values. We therefore used the accurate potential energy curve of the ground electronic state 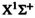
obtained by Coxon and Hajigeorgiou [62], who employed a direct potential fit (DPF) procedure, to ensure the accuracy of our line lists.

**FIGURE 5:**
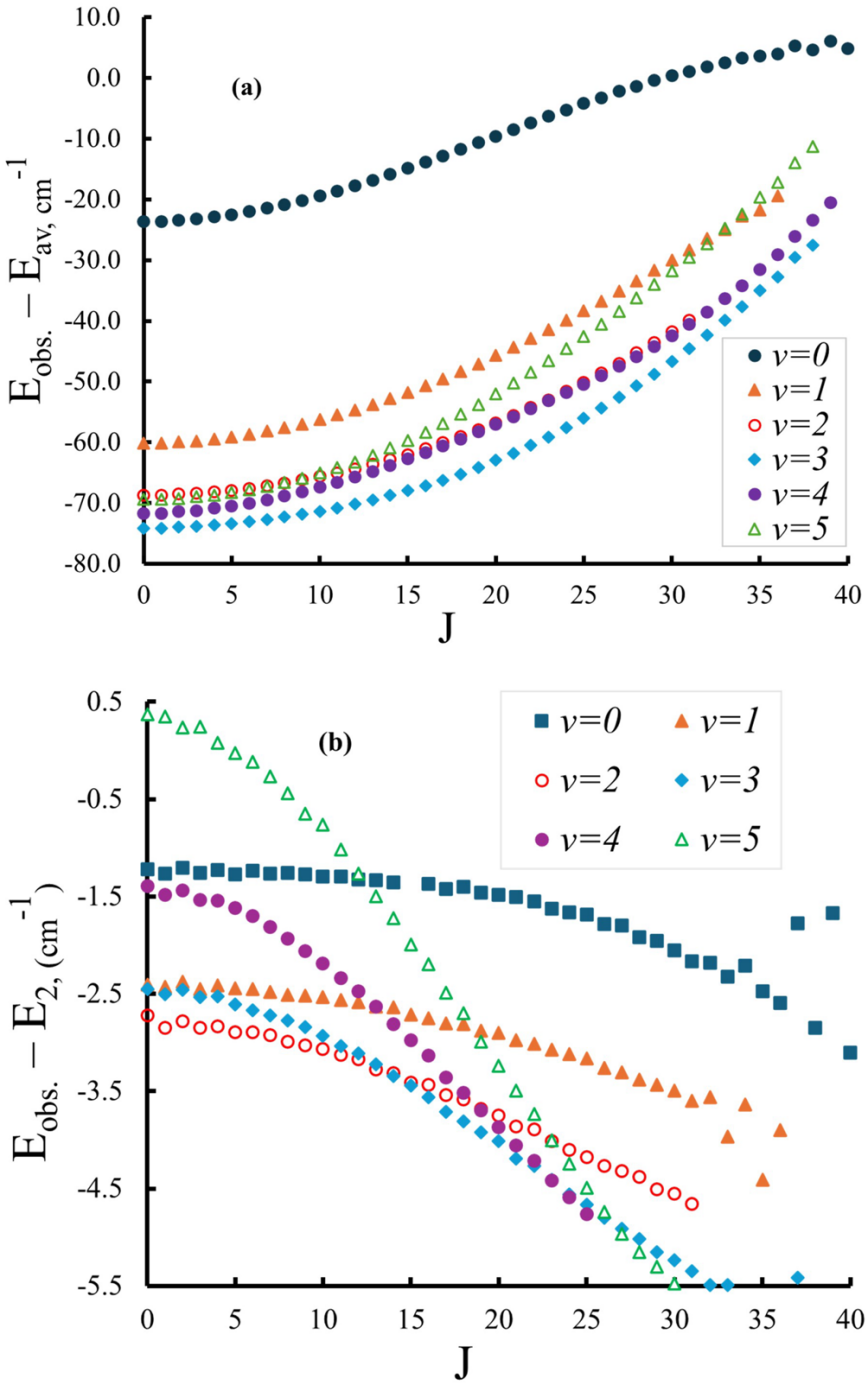
Observed minus calculated residuals for the 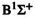 state using state‐averaged (upper panel) and two‐states‐averaged (lower panel). The residuals are presented as a function of J quantum number covering the vibrational levels up to v=5 with the energy range from $83,000\;{\text{cm}}^{-1}$ to $91,000\;{\text{cm}}^{-1}$. Observed term values were taken from Di Lonardo and Douglas [19].

Both 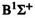 PECs obtained with two different methods of state average calculations, as mentioned in Section 3.1, were shifted to match the experimental value [19] of $T_{\text{e}}=84,783\;{\text{cm}}^{-1}$. Then, both curves were separately incorporated into the Duo program to compare the term values of the rovibrational energy levels obtained with each method. The energy levels of 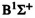 state using state‐averaged and two states‐averaged calculations are denoted as $E_{\text{av}}$ and $E_{2}$, respectively. Figure 5 provides an overview of the residuals of the 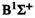 state using state‐averaged (panel a) and two‐states‐averaged (panel b) as a function of the total angular momentum quantum number J and covering vibrational levels up to v=5, which lie in the energy range $83,000-91,000\;{\text{cm}}^{-1}$. The residuals are the difference between the observed term values determined by Lonardo and Douglas [19] and our calculated term values. Comparing the results of the two panels as shown in Figure 5, the two‐state‐averaged rovibrational energy values turn out to be more accurate than state‐averaged ones, with all residuals below $5.\;5\;{\text{cm}}^{-1}$.

The 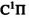 state was found to be best represented by an Extended Morse Oscillator (EMO) function [63] to facilitate the refinement of the experimental model. Using an EMO potential ensures an accurate dissociation limit and provides additional flexibility in the corresponding polynomial's degree around a reference position. The equation of EMO potential has the following form 
(1)

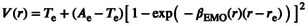

where $T_{\text{e}}$ is the electronic excitation energy (which for the ground state 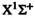 was set to zero), $A_{\text{e}}-T_{\text{e}}=D_{\text{e}}$ is the dissociation energy, $A_{\text{e}}$ is the corresponding dissociation limit, $r_{\text{e}}$ is the equilibrium internuclear bond distance of a PEC, and $\beta_{\text{EMO}}$ is the distance‐dependent exponent coefficient, defined as

(2) $$\beta_{\text{EMO}}\left(r\right)=\overset{N}{\underset{i=0}{\sum }}a_{i}\xi_{p}{\left(r\right)}^{i}$$

where N is the expansion order parameter, and $\xi_{p}$ is the Šurkus variable [64]. The Šurkus variable is defined as

(3) $$\xi_{p}\left(r\right)=\frac{r^{p}-r_{\text{ref}}^{p}}{r^{p}+r_{ref}^{p}}$$

where p is a non‐zero real number that serves as a variable parameter for precisely fitting the PECs, and $r_{\text{ref}}$ is a reference position ($r_{\text{ref}}=r_{\text{e}}$, by default).

The parameters $T_{\text{e}}$ and $r_{\text{e}}$ were set to 13.12 eV ($105,820\;{\text{cm}}^{-1}$) and 1.04 Å, respectively, as reported by Huber and Herzberg [39]. The electronic excitation energy $T_{\text{e}}$ and the dissociation energy $D_{\text{e}}$ of the ground state 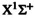 are reported as 0 and 6.12 eV (corresponding to $49,361\;{\text{cm}}^{-1}$), respectively, according to the same source. As a result, the asymptotic energy limit $A_{\text{e}}$ is simply:

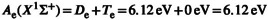

However, for the excited 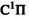 state, experimental values for both the asymptotic limit $A_{\text{e}}$ and the dissociation energy $D_{\text{e}}$ are not directly available. Nonetheless, these values can be estimated using known experimental data: The 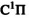 state correlates with the second dissociation limit, which lies at 10.199 eV (or $82,259\;{\text{cm}}^{-1}$) above the ground‐state minimum, as provided by NIST [48]. This value represents the electronic energy separation between the asymptotes of the 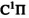 and $X^{1}\Sigma^{+}$ states. By adding this to the ground‐state asymptotic limit (6.12 eV), we obtain the asymptotic energy of the 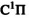 state:

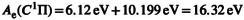

Consequently, the dissociation energy $D_{\text{e}}$ of the 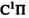 state was estimated to be 3.20 eV (or 25,809 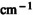) as follows:

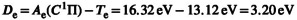

**FIGURE 6:**
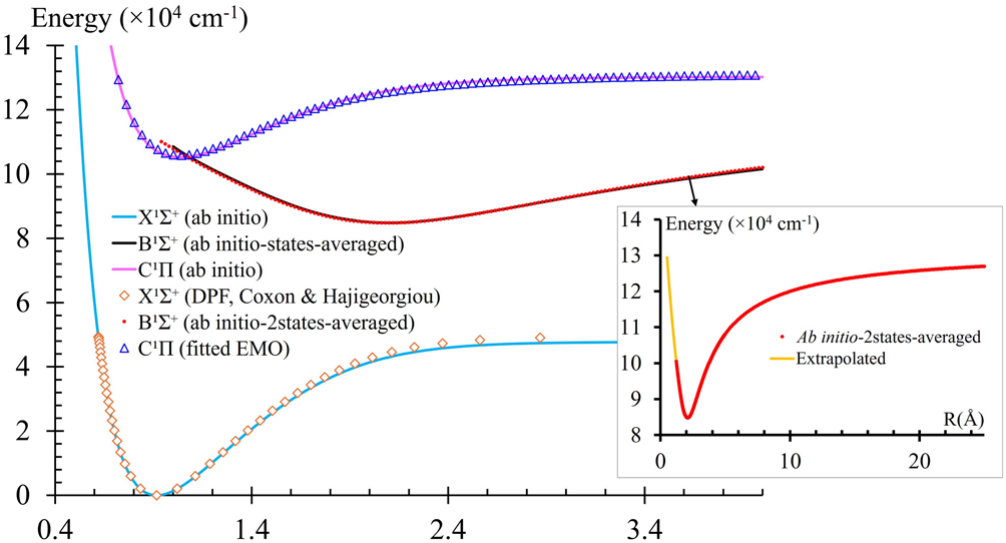
The calculated ab initio potentials and empirical/fitted EMO potentials of the $X^{1}\Sigma^{+}$, 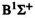, and 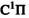 states for HF, in addition to the PEC of 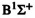 at the large internuclear distance.

The EMO expansion coefficients $a_{0}$ through $a_{4}$ were obtained by fitting the EMO function to the ab initio PEC data using the CurveExpert software [65]. Fixed and fitted parameters used in the EMO potential energy curve representation of the $C^{1}\Pi$ electronic state for the HF molecule are summarized in Table 3. A comparison between the empirical/fitted and ab initio PECs of the X 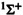, 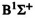, and 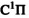 states of the HF molecule is shown in Figure 6. The ab initio PDMCs, TDMCs, and EAMCs, with the DPF [62] of the $X^{1}\Sigma^{+}$ PEC, the two‐states‐averaged of the 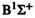 state, and the fitted EMO 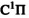 states, were directly introduced into Duo for line list calculations.

**TABLE 3:** Parameters, both fixed and obtained by fitting, used in the EMO potential energy curve representation of the ${\text{C}}^{1}\Pi$ electronic state of the HF molecule.

Category	Parameter	Value	Source
Fixed	$T_{\text{e}}$ (${\text{cm}}^{-1}$)	105,820	Huber and Herzberg [39]
	$r_{\text{e}}$ (Å)	1.04	Huber and Herzberg [39]
	$D_{\text{e}}$ (${\text{cm}}^{-1}$)	25,809	Calculated from experimental data
	p	2	Chosen value
Obtained after fitting	$a_{0}$	2.014273366201005	CurveExpert fit
	$a_{1}$	0.03074725969306062	
	$a_{2}$	0.7092070358504163	
	$a_{3}$	−0.8924745374962777	
	$a_{4}$	−1.002142797254693	

Line lists for the 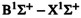 and 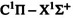 HF band systems were calculated by considering all rovibronic states and allowed transitions that satisfy the dipole selection rule ΔJ=0, ±1. The generated line lists cover wavenumbers up to $130,000\;{\text{cm}}^{-1}$ (wavelengths longer than 77 nm) and are presented in standard ExoMol format [66, 67] in the Supporting Information. They consist of a .states file, which contains the term energies and quantum numbers for each state, and a .trans file, which lists the Einstein A coefficients $A_{if}$ along with the numbers of the initial (i) and final (f) states. These numbers refer back to the corresponding entries in the .states file.

## 4 Simulated Spectra

In this section, we present the absorption spectra of HF using the generated line lists of 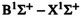 and 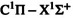 transitions and compare them with the available experimental work. All spectral simulations were performed using PGOPHER [68]. The general purpose of the PGOPHER program is to simulate rotational, vibrational, and electronic spectra (emission, absorption) at different temperatures from molecular line lists. PGOPHER offers a flexible graphical user interface that simplifies a wide range of processes involved in the simulation, interpretation, and fitting of molecular spectra. Its functionality includes the creation of Fortrat diagrams, detailed energy level visualizations, and the ability to overlay experimental spectra with simulated results for comparison. At the same time, the spectral simulations were cross‐checked with the ExoCross code [69]. ExoCross is a Fortran code for generating spectra (emission, absorption) at different temperatures and pressures from molecular line lists. ExoCross can simulate spectra for non‐local thermal equilibrium (non‐LTE) and compute lifetimes, cooling functions, specific heats, and other important physical properties. Both PGOPHER and ExoCross take input in ExoMol format.

### 4.1 Electronic Spectrum of HF

The electronic spectrum of hydrogen fluoride has been subject to numerous laboratory observations [13, 21, 22, 24, 70]. Hitchcock et al. [22] carried out experimental electron impact measurements to understand the molecule's photoabsorption properties. The investigations include the calculation of absolute cross sections obtained from various electronic transitions. Douglas and coworkers [18, 19, 20], focused on providing the optical absorption spectra of the 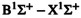 and 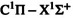 band systems. In the present work, we computed the 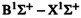 and 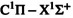 absorption spectra in terms of cross sections using the computed line lists obtained in Section 3.2. An energy resolution (FWHM) of 0.06 eV with grid bin size 0.124 meV and a temperature of T=673K were adopted to match the experimental conditions by Hitchcock et al. [22].

**FIGURE 7:**
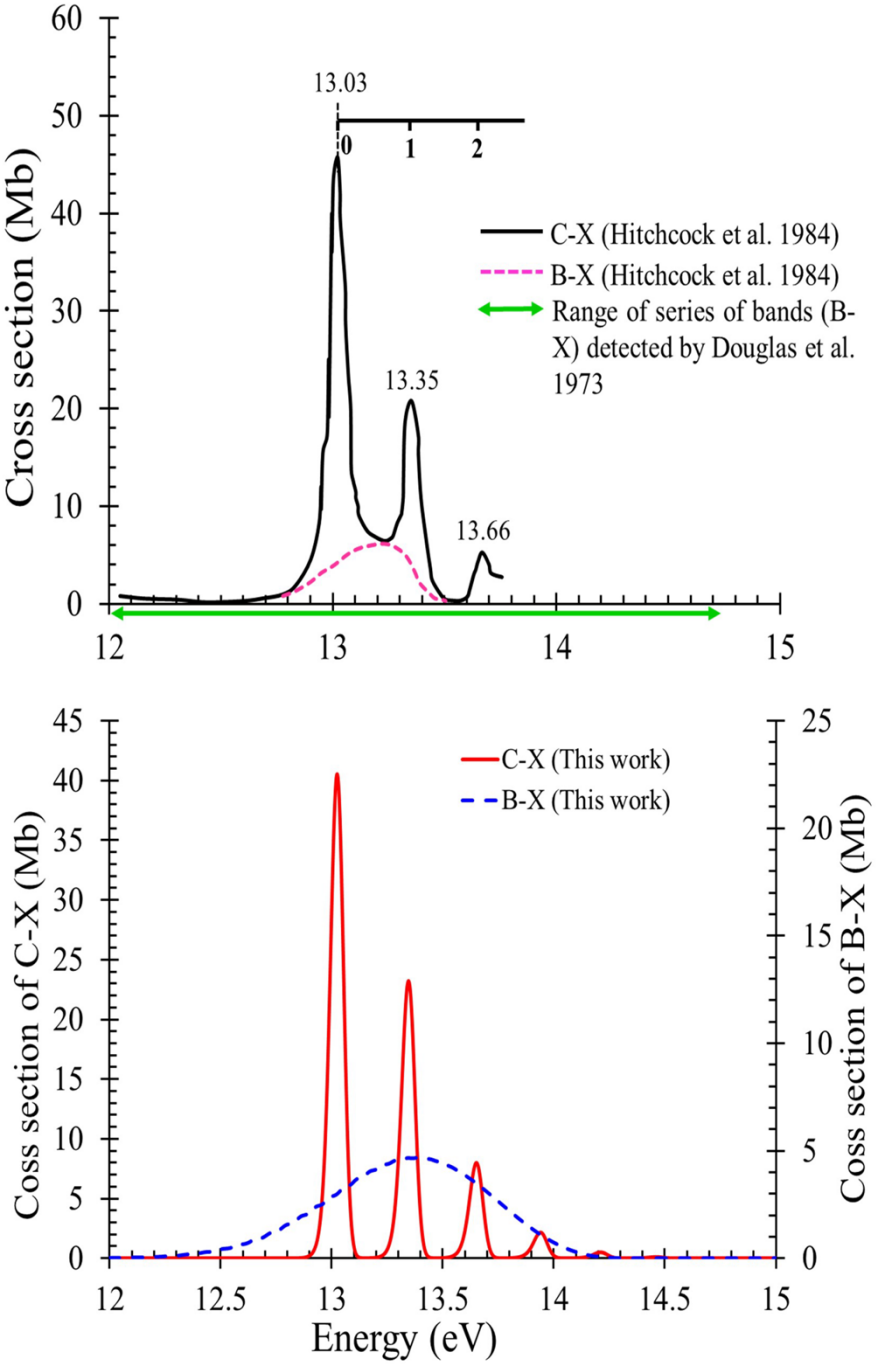
Comparison of photoabsorption cross section for 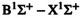 and 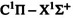 band systems deduced from observation by Hitchcock et al. [22] (upper panel) with our simulated spectra (lower panel).

Figure 7 illustrates a comparison of the 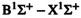 and 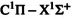 electronic photoabsorption cross section spectra observed by Hitchcock et al. [22] (upper panel) with the simulated spectra obtained in this work (lower panel).

The 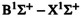 spectrum calculated in this work extends from 12 to 14.7 eV. This is in agreement with the spectroscopic investigations led by Di Lonardo and Douglas [18, 19], Douglas and Greening [20], who reported an extensive series of bands in the $X^{1}\Sigma^{+}\to B^{1}\Sigma^{+}$ system extending from 11.940 to 14.672 eV (range delimited by a green arrow in the upper panel of Figure 7). Hitchcock et al. [22] provide estimated cross sections for the 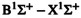 band system in the range 12.7 to 13.5 eV, as indicated by the pink dashed curve centered at 13.20 eV, on the same figure. The proposed position of the experimental peak is in accordance with our result, with both yielding a value of 13.35 eV. The value of the cross section at the peak positions differs however, where the experimental value is about $6\times 10^{-18}{\text{cm}}^{2}$/molecule while the value obtained in this work is less by about a factor of 1.28, with a cross section equal to $4.7\times 10^{-18}\;{\text{cm}}^{2}$/molecule. A similar discrepancy was observed with Nee et al. [71] and Pezzella et al. [41], who reported that the peak absorption cross section that they obtained for the 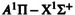 transition of HF was a factor of two lower than that given by Hitchcock et al. [22]. The authors proposed that the experimental data reported by Hitchcock et al. [22] may involve misassignments regarding transitions of different nature, specifically between continuum and discrete states. Additionally, as shown in Figure 7, the optical studies obtained by Di Lonardo and Douglas [18, 19], Douglas and Greening [20] reported an extensive series of bands extending from 11.940 to 14.672 eV, which are attributed to the 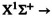 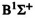 excitation, this extended part is not detected by the energy loss spectrum reported by Hitchcock et al. [22]. According to Hitchcock and Brion [21], the absence of these features in the energy loss spectrum is not fully understood. They assign this weak signal to factors such as contributions from other excitations, the inherently weak intensity of the transition, or differences in the excitation mechanisms between optical absorption and fast electron impact.

As can be seen in the upper and lower panels of Figure 7, peak positions for the 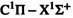 transition ($v^{\prime }=0,1,2$) agree exceptionally well with Hitchcock et al. [22]. The values of the cross sections are also in accordance with the investigated peaks. Table 4 provides a detailed comparison between observed and calculated peak positions of the studied 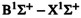 and 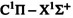 transitions. The experimental data is taken from Hitchcock et al. [22], while calculated values include results from this present work as well as available theoretical results in the literature. Excellent agreement is observed when comparing the peak positions of the 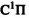 ($v^{\prime }=0,1,2,3$) states with those experimentally obtained, yielding zero residuals. In addition, Table 4 reports close alignment between this work's peak positions and the previous theoretical results due to Bettendorff et al. [29], Dunning Jr. [32], Bender and Davidson [72], Segal and Wolf [73], and Pezzella et al. [41] for the studied transitions.

**TABLE 4:** Comparison of observed and calculated excitation energies, in eV for the and states; residuals are reported as Obs.−Calc., in eV. The observed values are taken from Hitchcock et al. [22]. The character, vibronic progression, and peak positions are provided for each electronic state.

State	Character	Transition	Obs.	Calc. [Ref.]	Obs.−Calc.
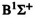	Diffuse	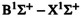	13.20	13.35 [This work]	−0.15
				13.99 [72]	−0.79
				13.7 [32]	−0.5
				13.84 [73]	−0.64
				13.40 [29]	−0.2
				13.20 [41]	0.00
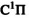	Sharp	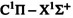			
		0−0	13.03	13.03 [This work]	0.00
				17.40 [72]	−4.37
				13.52 [73]	−0.49
				13.07 [29]	−0.04
				13.03 [41]	0.00
		1−0	13.35	13.35 [This work]	0.00
		2−0	13.66	13.66 [This work]	0.00
		3−0	13.94	13.94 [This work]	0.00

As shown in Table 4, there is a significant difference between our excitation energy values and those obtained by Bender and Davidson [72], in particular for the 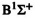 and 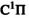 states. In that work, the low‐lying electronic states of the hydrogen fluoride molecule were investigated using extensive configuration interaction with a limited basis set. As discussed by Hitchcock et al. [22], who obtained a similar standard deviation of $4.37\;{\text{cm}}^{-1}$ to ours, this basis does not include diffuse functions and therefore cannot properly describe the B and C states, which include contributions from Rydberg states.

### 4.2 Photoabsorption Rovibronic Spectrum of HF

#### 4.2.1 Band

**FIGURE 8:**
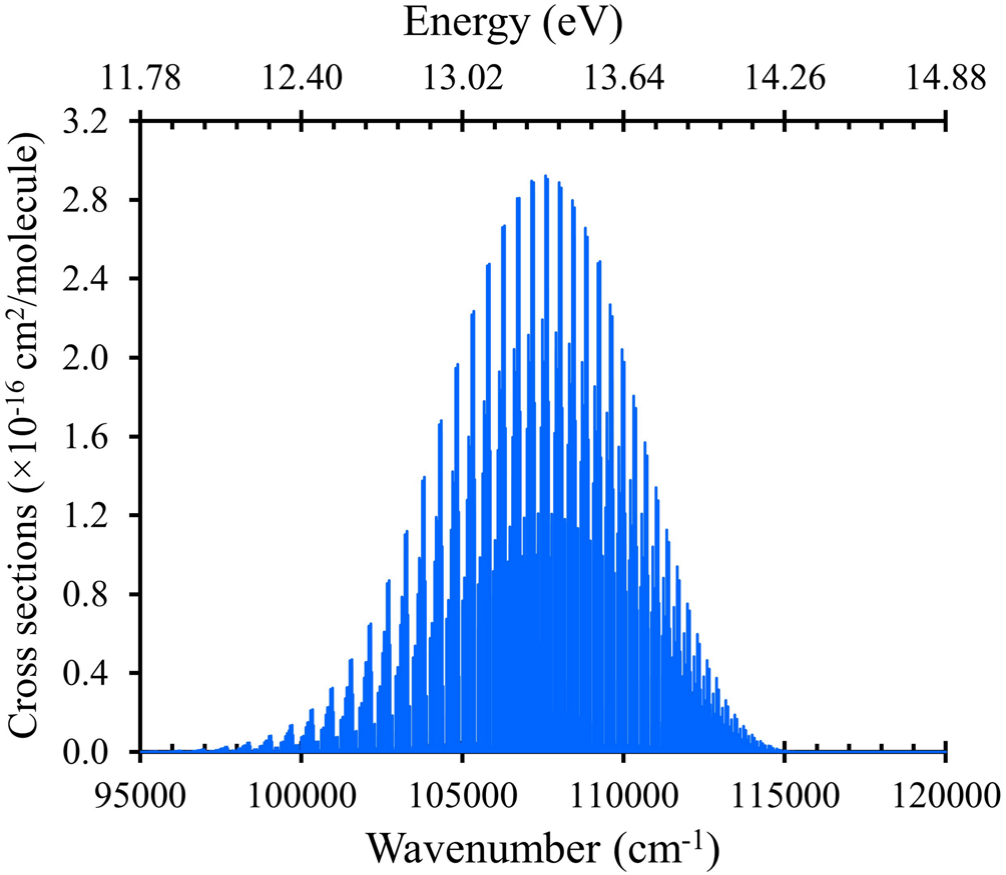
The simulated absorption spectrum of the 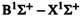 cross sections in the range $95,\;000-120,\;000\;{\text{cm}}^{-1}$. The temperature is T=300K with a Gaussian line profile with a full width at half maximum (FWHM) of $1.0\;{\text{cm}}^{-1}$.

A representation of the HF 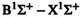 rovibronic absorption spectrum in terms of cross section is shown in Figure 8, using our line lists of transitions in the range $95,000-120,000\;{\text{cm}}^{-1}$ (corresponding to 83.3–105.3 nm). The temperature is set at T=300K with a Gaussian line width of $1.0\;{\text{cm}}^{-1}$.

**FIGURE 9:**
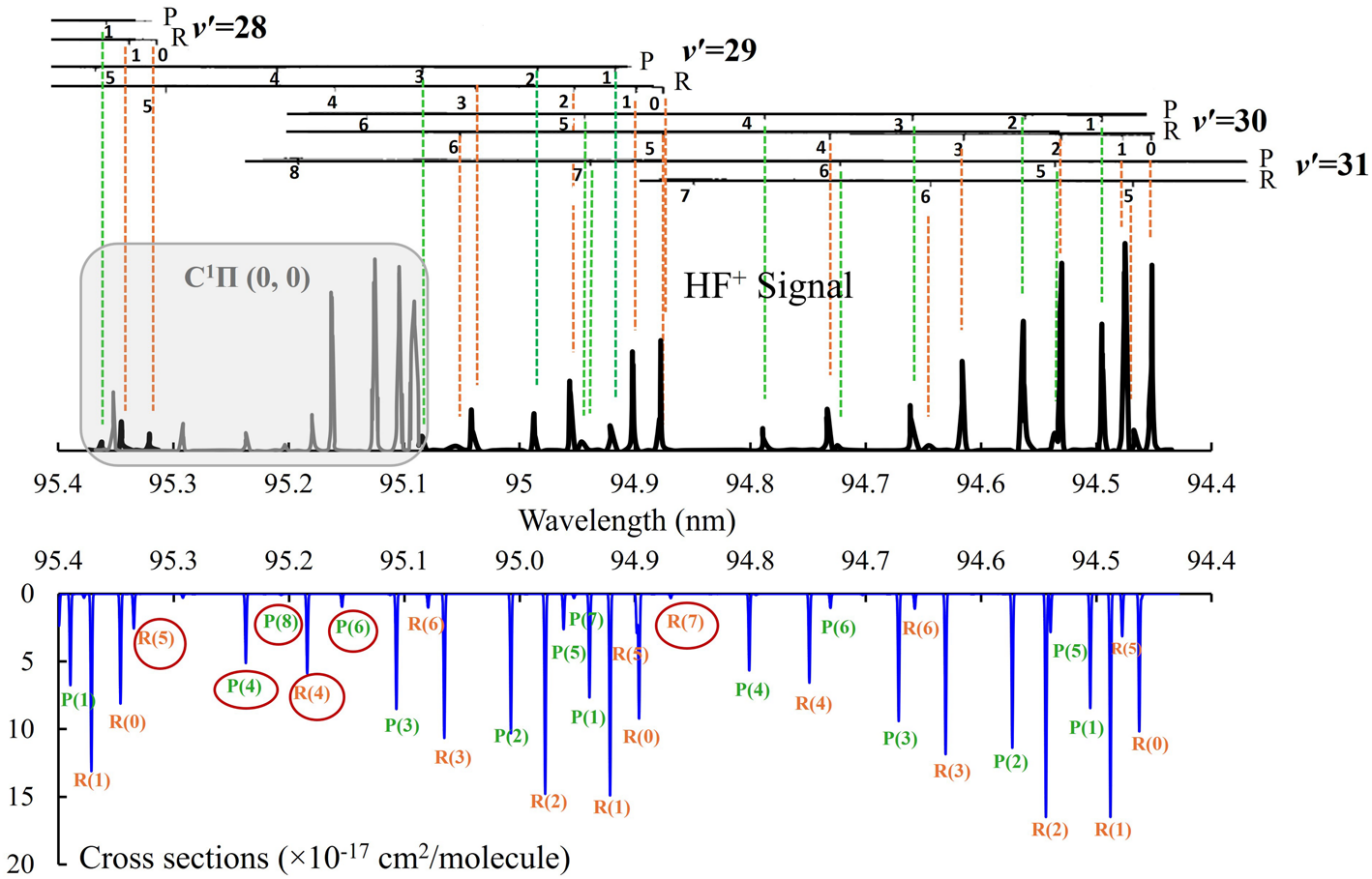
Comparison between the bands of the 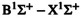 spectrum of HF observed by Tashiro et al. [23] (upper) with this work's simulated spectrum (lower). The spectral lines highlighted with circles represent features that were not reported experimentally, but are identified theoretically in both our calculations and Tashiro's energy level (term) diagram. The unassigned lines in Tashiro's diagram correspond to the theoretically predicted features circled in our simulation. A more detailed analysis of the 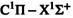 transition is presented in Figure 13.

A portion of this spectrum has been experimentally investigated in the extreme ultraviolet (XUV) regime by Tashiro et al. [23], in the wavelength range 94.4–95.4 nm. The experiment was performed using 1 XUV + 1 UV REMPI, permitting the observation of lines corresponding to transitions from the ground state $X^{1}\Sigma^{+}$ $v^{\prime \prime }=0$ to the 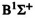 state $v^{\prime }=28$, 29, 30, and 31. Figure 9 shows a comparison between the four bands: 28‐0, 29‐0, 30‐0, and 31‐0 of the 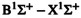 spectrum of HF observed by Tashiro et al. [23] (upper panel) and that computed using our line lists assuming a temperature of 300 K. A Gaussian profile line width of $1.5\;{\text{cm}}^{-1}$ is used (lower panel). A good overall match is observed between the two spectra in the P‐ and R‐branches, with minor redshifts appearing in some bands of the lower panel. It was previously [19, 23, 73] postulated that the observed vibrational levels of 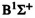 state starting from v=24 are perturbed and that such perturbations might arise from two different Rydberg states (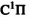 and 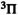). The first serious perturbation, as analyzed by Lonardo et al. [19], was caused by the interaction of the 27‐0 band and weak band system 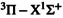. Above v=27, many severe perturbations occur due to the mixing of the bands of the 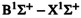 system with a portion of strong 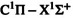 Rydberg bands. Consequently, the difference in the positions of the observed 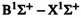 transition and our calculated one may be due to several factors, including the severe perturbations that arise from the mutual interaction between 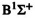 and 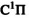 states. On the other hand, the assignments of the 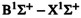 spectrum of HF reveal lines not observed experimentally but predicted by our calculations and the energy‐level diagram of Tashiro et al. [23].

**FIGURE 10:**
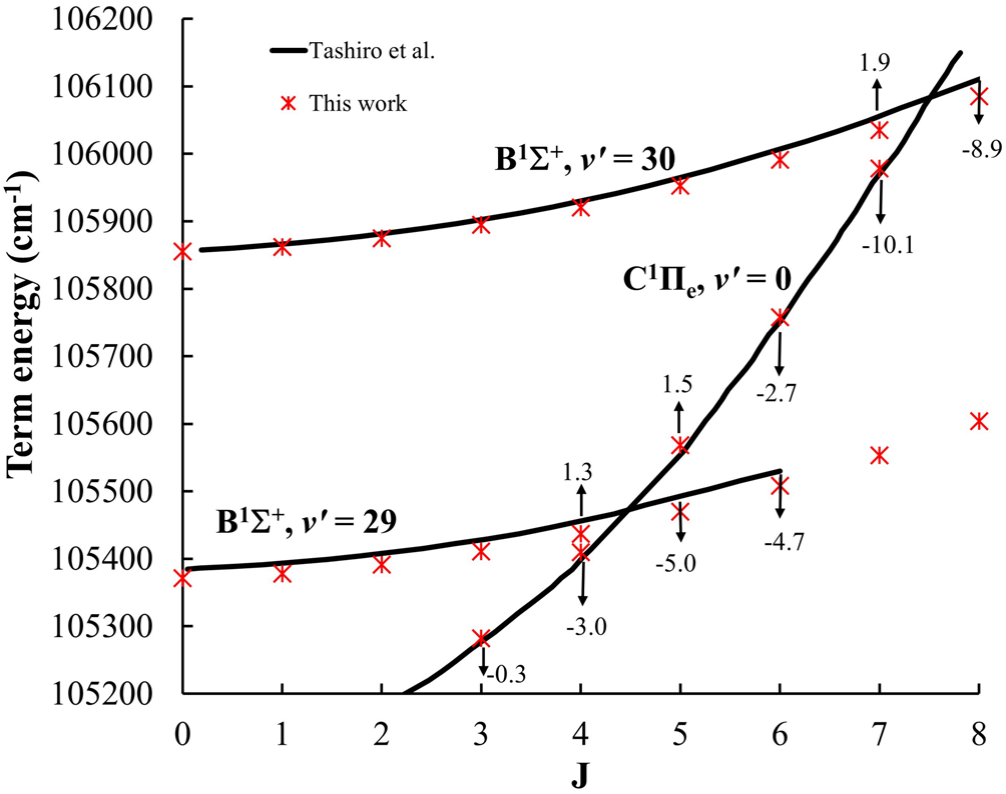
A comparison of our calculated term energies of the B state ($v^{\prime }=29$ and 30), and of the e‐doublet component of the C state, with the values reported by Tashiro et al. [23], plotted as a function of J. The solid lines represent energies calculated from a fitting of unperturbed lines. The arrows indicate the energy shifts (in ${\text{cm}}^{-1}$) of the observed levels relative to those calculated ones.

Figure 10 presents a comparison of our calculated term energies of the 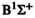 state for $v^{\prime }=29$ and 30, and of the e‐doublet component of the C state $v^{\prime }=0$, with the values reported by Tashiro et al. [23], plotted as a function of J. The solid lines represent energies calculated from a fitting of unperturbed lines. The arrows indicate the energy shifts (in ${\text{cm}}^{-1}$) of the observed levels relative to those calculated ones. For both the B and C states, the differences between the observed and the calculated unperturbed values agree with a maximum shift of less than 10 ${\mathrm{cm}}^{-1}$. Tashiro et al. [23] attributed this shift to perturbations between the B and C states. In our comparison, a similar deviation is observed in our results.

A REMPI rotationally resolved spectrum represents the collected mass‐selected ion signal as a function of the XUV wavelength. The spectrum calculated in this work and the mass‐selected (1 + 1) REMPI spectrum share the same underlying transition energies, including those corresponding to intermediate states accessed during the process, leading to similar spectral line positions [74]. REMPI involves a second photon for ionization, meaning that spectral intensity depends on the product of both the initial transition probability and the ionization cross section from the intermediate state. This is different from our calculated single‐photon band intensities. This difference causes the variations in peak intensities between the theoretical and experimental spectra shown in Figure 9.

#### 4.2.2 Band

**FIGURE 11:**
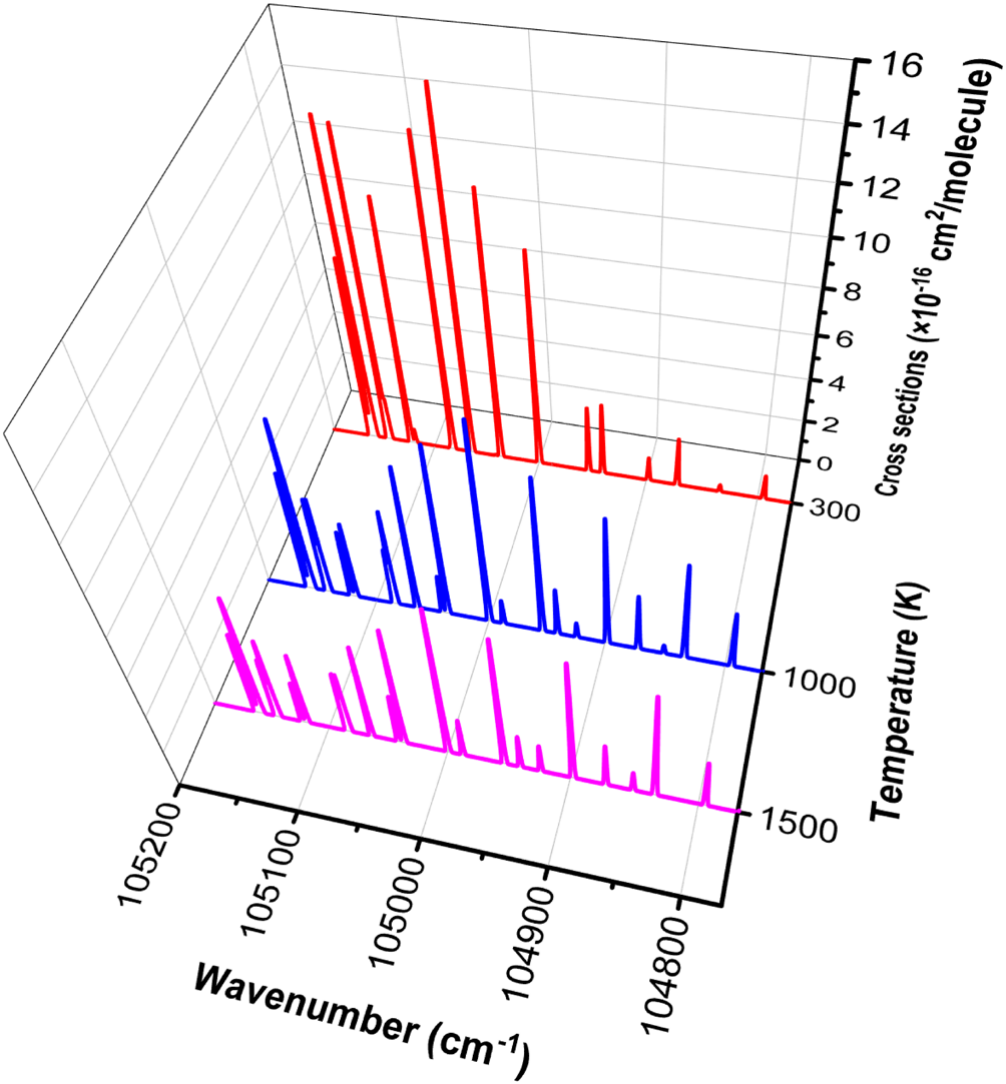
Temperature dependence of HF cross sections obtained using our line lists of 0‐0 band $C^{1}\Pi$‐$\;X^{1}\Sigma^{+}$ transition using a Gaussian profile with $\text{HWHM}=\;1{\text{cm}}^{-1}$.

An overview of the HF rovibronic 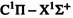 (0‐0) band absorption cross section spectrum at different temperatures T=300 K, 1000 K, and 1500 K is shown in Figure 11.

**FIGURE 12:**
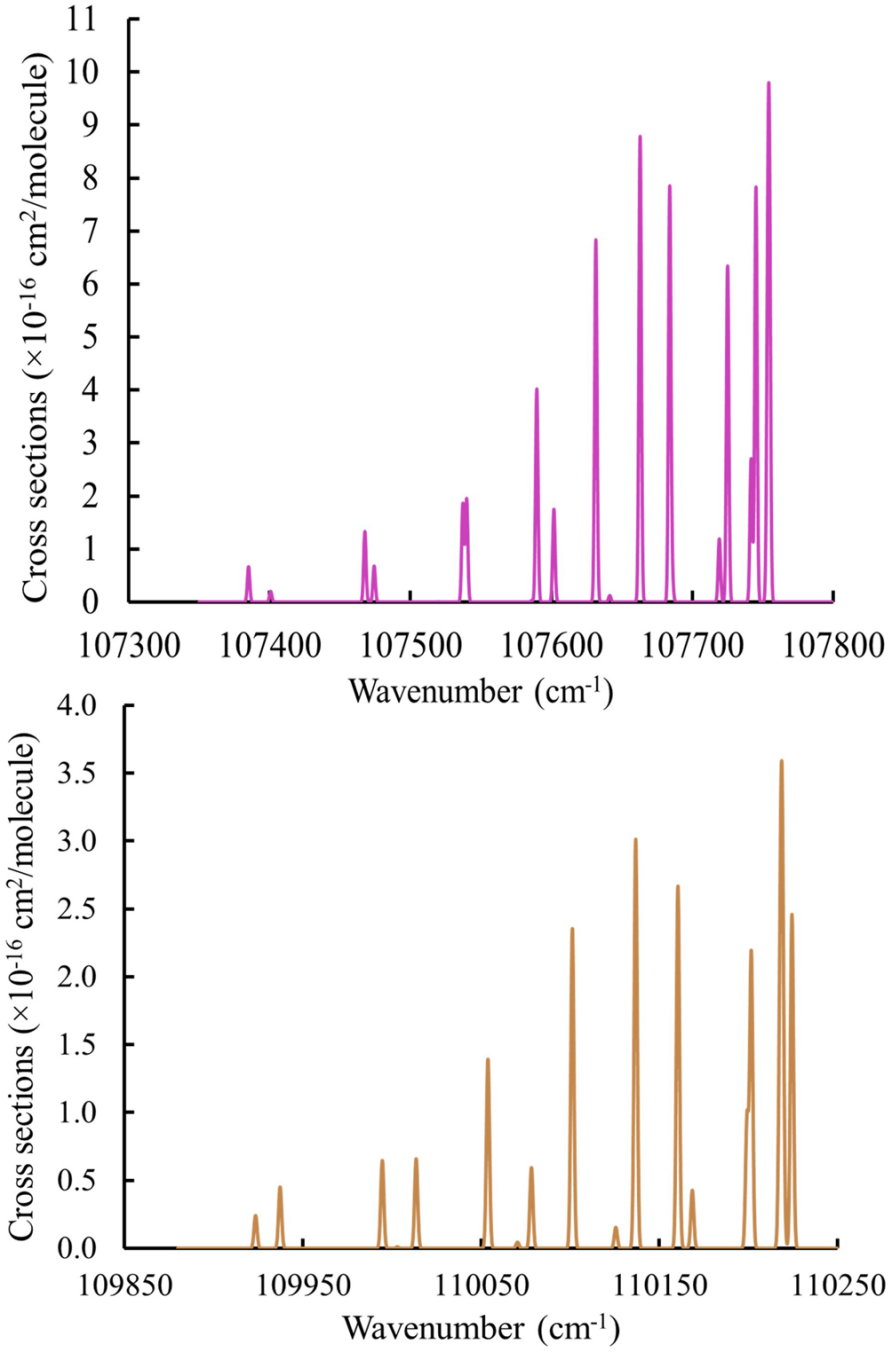
Simulated absorption spectra of the 1‐0 (upper panel) and 2‐0 band (lower panel) 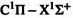 cross sections obtained using our line lists of transitions. The spectrum was simulated at T = 300 K with a Gaussian profile with $\text{HWHM}=1\;{\text{cm}}^{-1}$.

**FIGURE 13:**
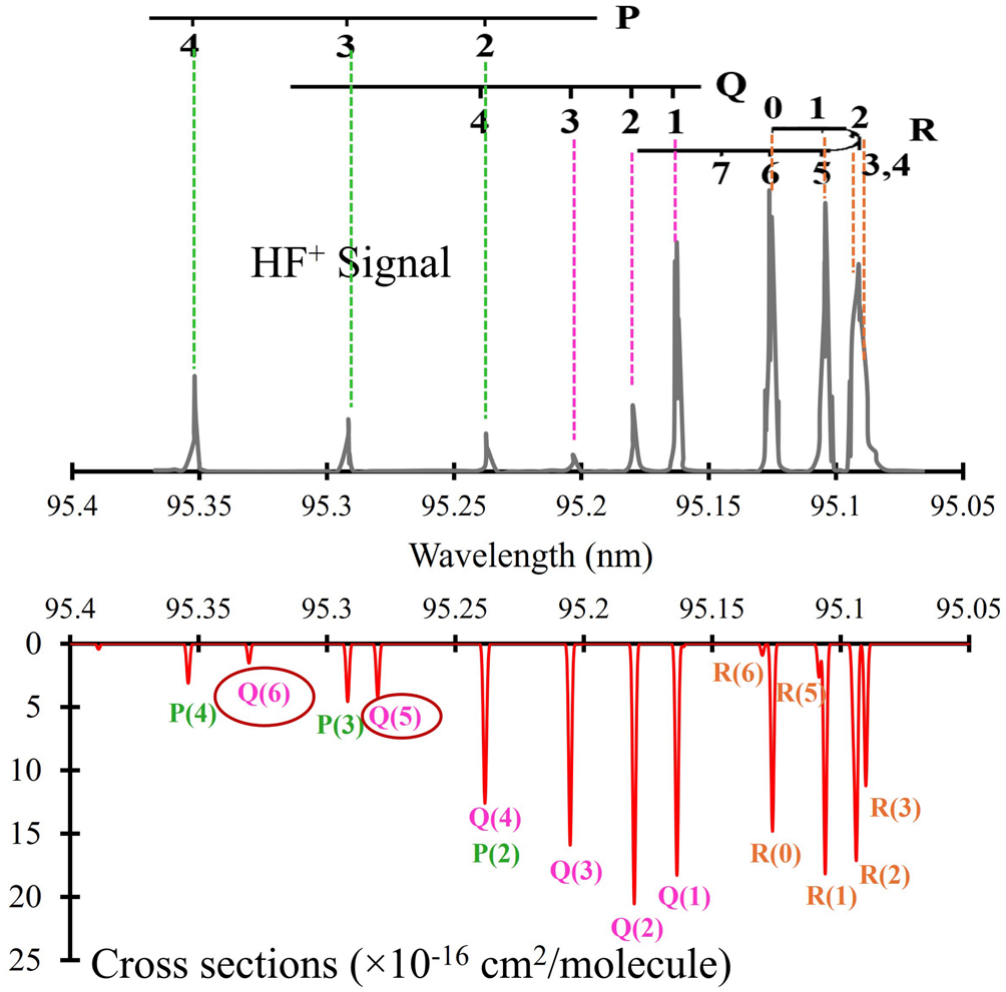
Comparison between the 0‐0 band of the ${\text{C}}^{1}\Pi$ spectrum of HF observed by Tashiro et al. [23] (upper) and our simulated spectrum using our line lists (lower), assuming a temperature of 300 K. A Gaussian profile line width of $1.5\;{\text{cm}}^{-1}$ is used. The spectral lines highlighted with circles represent features that were not reported experimentally but are identified in our calculations.

Figure 12 presents an overview of rovibronic spectra for the 1‐0 and 2‐0 vibrational bands at T=300K. To the best of our knowledge, the only observation of the 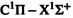 band system was reported by Tashiro et al. [23] in the wavelength range 94.4–95.0 nm corresponding to transitions from the ground state 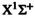 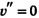 to the 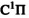 state 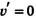. Figure 13 compares a portion of the absorption spectrum of the (0‐0) band for 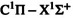 observed by Tashiro et al. [23] with our spectrum, which is simulated at the same temperature as the experimental one. It shows a good correspondence between the wavelength and the assignments for each branch. Some lines identified in our calculations were not reported experimentally, as shown in Figure 13. Table 5 lists the line positions of the HF molecule 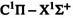 (0‐0) band system, the observed values [23], and the Obs. – Calc. residues are reported for R‐, Q‐, and P‐branches. Tashiro et al. [23] reported the deviations between the observed line position of C‐X band and those calculated from the fitting of C‐X Q branch transitions. These maximum deviations for the R‐, Q‐, and P‐branch transitions are approximately 7.3, 0.16, and 10.0 ${\text{cm}}^{-1}$, respectively. A comparison between the observed spectral line positions from experimental measurements [23] and the values calculated in this work reveals that the validated Q‐branch lines exhibit the highest accuracy, with deviations (Obs. – Calc.) remaining below $4.54\;{\text{cm}}^{-1}$. For the P‐ and R‐branch transitions, the calculated line positions show good agreement with experimental data at low rotational quantum numbers (J), where deviations are less than $0.9\;{\text{cm}}^{-1}$ (up to J=3 for the R‐branch and up to J=5 for the P‐branch). However, the errors increase beyond this threshold as J due to the various perturbations.

**TABLE 5:** Line positions (in ${\text{cm}}^{-1}$) of the HF molecule ${\text{C}}^{1}\Pi -{\text{X}}^{1}\Sigma^{+}$ (0‐0) band system compared with the observed values.

J	R(J)			Q(J)			P(J)		
	Obs.	Calc.	Obs.‐Calc.	Obs.	Calc.	Obs.‐Calc.	Obs.	Calc.	Obs.‐Calc.
0	105,123.09	105,123.02	0.07						
1	105,146.09	105,145.73	0.36	105,082.05	105,081.91	0.14			
2	105,159.88	105,159.14	0.74	105,064.12	105,063.56	0.56	104,999.79	104,999.74	0.05
3	105,162.54	105,163.20	−0.66	105,037.37	105,036.01	1.36	104,940.95	104,940.43	0.52
4	105,163.76	105,157.84	5.92	105,001.54	104,999.26	2.28	104,872.96	104,872.08	0.88
5	105,147.94	105,143.00	4.94	104,957.84	104,953.30	4.54	104,794.19	104,794.73	−0.54
6	105,121.15^a^	105,118.59	2.56	104,905.96^a^	104,898.10	7.86	104,714.40	104,708.41	5.99
7	105 096.74	105 084.56	12.18				104 618.32	104613.16	5.16
8		105,040.81					104,511.57	104,509.00	2.57
9	105,007.15^b^	104,987.29	19.86						
10	104,987.83^b^	104,923.91	63.92						

^a^ Line positions are taken from Douglas and Greening [20].

^b^ The assignment is uncertain, as mentioned by Tashiro et al. [23].

A similar trend was reported by Tashiro et al. [23], who attributed these higher‐level discrepancies to significant perturbations between the ${\text{B}}^{1}\Sigma^{+}$ and ${\text{C}}^{1}\Pi$ states. This behavior can be understood in terms of the selection rules for electric dipole transitions [75]: Q‐branch transitions occur between states with opposite parity (*e*/*f*), whereas P‐ and R‐branch transitions involve states of the same parity. In the case of the 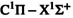 band system, the Q‐branch arises from transitions between the *f*‐parity components of the Λ‐doublets in the ${\text{C}}^{1}\Pi$ state and the *e*‐parity component of the ground state. As proposed by authors [23, 76], these Q‐branch lines are largely unaffected by the ${\text{B}}^{1}\Sigma^{+}$ state, which leads to acceptable values of the Q branch referred to in Table 5. On the other hand, the *e*‐parity Λ‐doublet components of the ${\text{C}}^{1}\Pi$ state, which contribute to the P‐ and R‐branch transitions, are subject to more significant perturbations, especially at the higher rotational levels. These perturbations are the cause of the larger values of the “obs‐calc” results referred to in the same table.

### 4.3 Lifetimes

Lifetimes of individual rovibronic states are also computed as part of the Duo calculations, based on the generated line list files, following the methodology of Tennyson et al. [77]. The total radiative lifetimes $\tau_{i}$ of the excited rovibronic states in the B and C electronic states were obtained by summing over all spontaneous emission rates from each initial state i to all energetically accessible lower states f:

(4) $$\tau_{i}={\left(\sum_{f}A_{if}\right)}^{-1}.$$

These lifetimes were computed using the ExoCross program [69].

**FIGURE 14:**
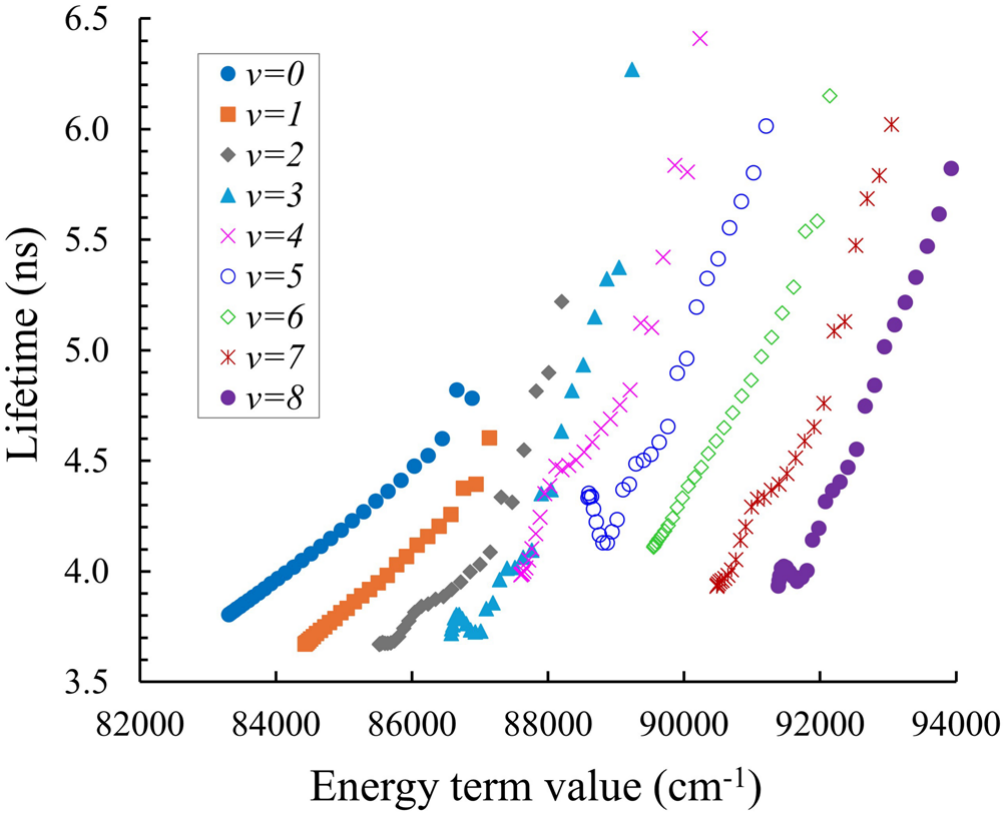
Lifetimes of the vibration–rotation states of HF in the 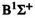 state. Curves correspond to states with increasing rotational quantum number J, plotted as a function of energy.

**FIGURE 15:**
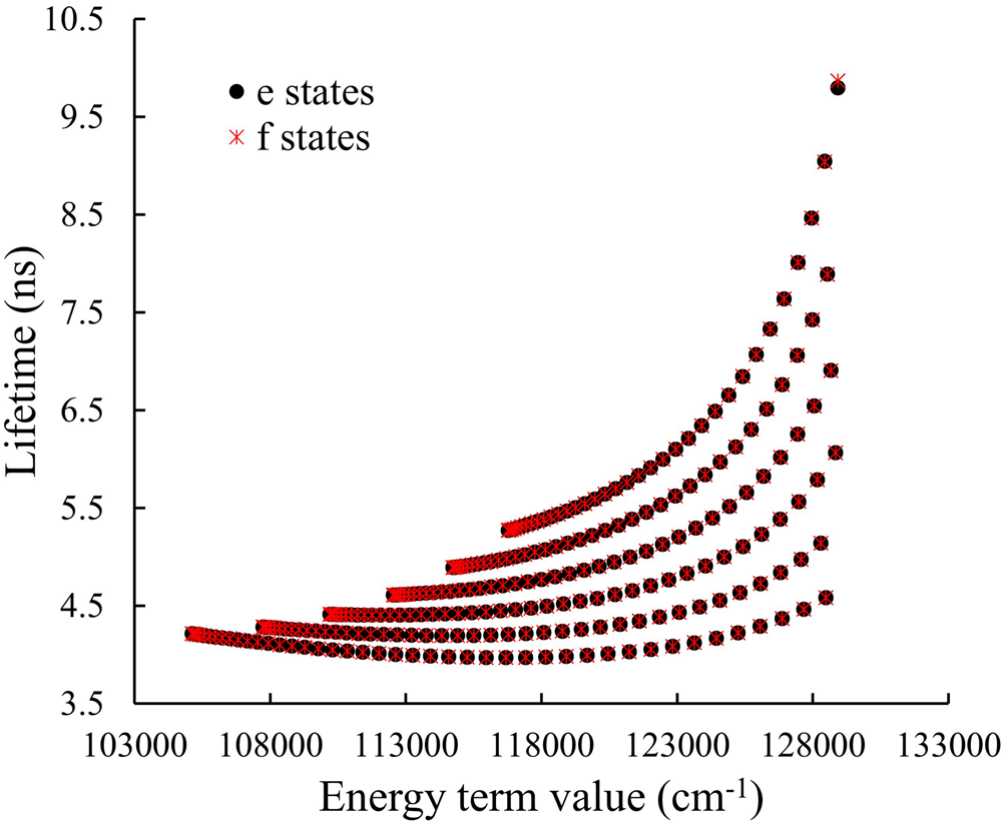
Lifetimes of the vibration–rotation (e and f parity) states of HF in the 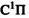 state for vibrational levels v=0, v=1 up to v=5. The curves correspond to states with increasing rotational quantum number J, plotted as a function of energy.

Figure 14 shows the lifetimes of the vibration–rotation states of HF in the 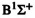
state as a function of the term energy values. The lowest states (v=0, small J) of the ${\text{B}}^{1}\Sigma^{+}$ state have shorter lifetimes. Figure 15 presents the lifetimes of the vibration–rotation (e and f parity) states of HF in the 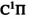 state for vibrational levels v=0,1,…,5. In general, these lifetimes change gradually with increasing vibrational quantum number v and rotational quantum number J, with higher excitations in either mode leading to shorter lifetimes. For all values of J, there appears to be no dependence on the spin component.

To the best of our knowledge, experimental radiative lifetimes for the ${\text{B}}^{1}\Sigma^{+}$ and 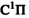 electronic states of HF have not yet been reported. The values presented here may therefore serve as useful reference data for experimentalists and guide future investigations. In contrast, theoretical radiative lifetimes for these states have been reported by Huang et al. [36] and Liu et al. [37], who calculated lifetimes for individual transitions using the LEVEL program [78]. Our approach differs in that the lifetimes were obtained with ExoCross [69] based on Duo calculations, where the resulting values represent the total radiative lifetime, including contributions from all three allowed photon polarizations (t=−1,0,+1). Consequently, there is a factor of three between these two methodologies. To enable a direct comparison between our results and the previously reported theoretical values, we therefore multiply our calculated radiative lifetimes by a factor of three. Table 6 compares our calculated radiative lifetimes for selected vibrational levels (up to $v^{\prime }$ = 10) for the 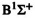 and 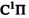 states with previously published theoretical values. For the 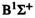 state, the values of the Einstein coefficients are small for the lower vibrational levels $v^{\prime \prime }$ due to the small Franck‐Condon factors between these states and the ground state, which are associated with the large shift between minima of these potential energy curves.

**TABLE 6:** Radiative lifetimes of the transitions from and to the ground X  of HF (in ns): results of this work compared to theoretical values by Liu et al. [37] and Huang et al. [36].

Transition	Ref.	max. $v^{\prime \prime }$	Radiative lifetimes (ns)										
			$v^{\prime }=0$	$v^{\prime }=1$	$v^{\prime }=2$	$v^{\prime }=3$	$v^{\prime }=4$	$v^{\prime }=5$	$v^{\prime }=6$	$v^{\prime }=7$	$v^{\prime }=8$	$v^{\prime }=9$	$v^{\prime }=10$
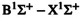	This work	21	11.42	11.02	11.01	11.22	11.96	13.06	12.35	11.81	11.88	11.71	11.37
	Liu et al. [37]	21	12.47	12.34	11.80	11.77	12.09	13.27					
	Huang et al. [36]	26	22.46	21.58	20.98	22.18	22.56	25.40	24.39	23.27	23.69	23.13	
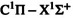	This work	18	12.65	12.85	13.24	13.83	14.67	15.82	17.34	19.37	22.05	25.59	30.31
	Liu et al. [37]	11	11.89	11.78	12.13	12.63	13.27	13.74					

Table 6 shows that the radiative lifetimes for 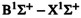 transition ($v^{\prime }\leq 10$) determined in this work are in accordance with those reported by Liu et al. [37] and are consistently shorter by about 10 ns (roughly a factor of two) compared to the values of Huang et al. [36]. This discrepancy is most likely due to a small difference between our TDMC and that of Huang et al. [36], as well as to differences in the corresponding transition energies. In the case of the 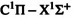 transition, our calculated lifetimes are close to the results of Liu et al. [37], which, to the best of our knowledge, represent the only previously available data for this transition. For the purpose of comparison, the authors have also calculated the radiative lifetimes of the lower vibrational levels for both 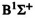 and 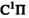 states using the same method (LEVEL 11) and considered the same maximum ground vibration level $v^{\prime \prime }$ as studied by Huang et al. [36] and Liu et al. [37]. The obtained lifetimes are 11.40 ns (for $v^{\prime }=0$) in the 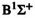 state and 12.63 ns (for $v^{\prime }=0$) in the 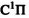 state. The results also compare reasonably well with those of the remaining vibrational levels up to $v^{\prime }=10$. The deviation probably arises from the slight difference in TDMCs used.

## 5 Conclusions

This study presents a comprehensive ab initio spectroscopic calculation for the low‐lying singlet and triplet states of the HF molecule. A complete set of PECs, PDMCs, TDMCs, and EAMCs was investigated using the following method with suitable basis sets: MRCI + Q/aug‐cc‐pV5Z and MRCI + Q/aug‐cc‐pV5Z‐DK. The PECs of the states incorporated in our studied transition systems were refined by fitting the ab initio curves to the experimental data. This refinement improves the accuracy of rovibrational energy levels, line lists, and spectral simulations of previously published results.

The refined curves are then used as an input in the Duo nuclear motion program to compute the rovibrational energy levels for the $X^{1}\Sigma^{+}$, 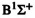, and 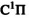 states and the line lists for the 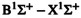, 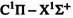 transitions. Using the obtained HF line lists, rovibronic spectral simulations were performed at different temperatures using the PGOPHER program (cross‐checked with the ExoCross code). Moreover, the vibrational radiative lifetimes ($\tau_{v^{\prime }}$) for the 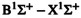 and 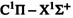 systems are calculated and compared with data published in the literature, yielding a positive agreement. Additionally, vibrational constants for the excited singlet and triplet states were predicted for future studies.

The PECs are through empirical refinement, (i) introducing couplings to the 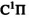 state to allow the perturbations to be modeled, (ii) providing updated vibrational radiative lifetimes, (iii) producing accurate rovibrational term values, line lists by incorporating an accurate ground state curve and simulated spectra, and (iv) assigning transition lines that could not be detected experimentally for B and C states. These improvements are crucial for interpreting the spectral signatures and provide a foundation for further research on the HF molecule.

## Conflicts of Interest

The authors declare no conflicts of interest.

## Supporting information




**Figure S1:** The initial and fitted/interpolated transition dipole moment curves for the B–X and C–X transitions.

## Data Availability

All data are available in the article or Supporting Information. The data that supports the findings of this study are available in the Supporting Information of this article.
