# Supplementary material for: First Principles Rovibronic Absorption Spectra of HF Molecule
Source: J Comput Chem. 2026 Feb 24;47(6):e70317. doi: 10.1002/jcc.70317 (PMC12930379; doi:10.1002/jcc.70317)
Supplement: Supplementary file 1 — Figure S1: The initial and fitted/interpolated transition dipole moment curves for the B–X and C–X transitions. [file JCC-47-0-s001.zip › TS1.docx]

**Table TS1: The table presents a comparison between experimental values and the present theoretical calculations using different basis functions**

| **States** | **Ref.** | **Basis functions** | **R_e_**  **(Å)** | $\boldsymbol{\Delta}\boldsymbol{R}_{\boldsymbol{e}}$  **(Å)** | **T_e_**  **(cm^-1^)** | $\boldsymbol{\Delta}\boldsymbol{T}_{\boldsymbol{e}}$  **(cm^-1^)** | **ω_e_**  **(cm^-1^)** | $\boldsymbol{\Delta}\boldsymbol{\omega}_{\boldsymbol{e}}$  **(cm^-1^)** | **B_e_**  **(cm^-1^)** | $\boldsymbol{\Delta}\boldsymbol{B}_{\boldsymbol{e}}$  **(cm^-1^)** |
| --- | --- | --- | --- | --- | --- | --- | --- | --- | --- | --- |
| **X^1^Σ^+^** | **[1]** | **Exp.** | **0.91680_8_** |  | **0** |  | **4138.32** |  | **20.9557** |  |
|  | This work | spd functions* | 0.917 | *-0.0002* | 0 | - | 4098.3 | *40.02* | 20.96 | 0.0043 |
|  | This work | spd functions** | 0.914 | *0.0028* | 0 |  | 4113.8 | *24.52* | 21.08 | -0.1243 |
|  | This work | Full functions* | 0.916 | *0.0008* | 0 |  | 4178.7 | *-40.38* | 21.06 | *0.1043* |
| **B^1^Σ^+^** | **[1]** | **Exp.** | **2.0908_6_** |  | **84 776.65** |  | **1159.18** |  | **4.0291** |  |
|  | This work | spd functions* | 2.099 | *-0.0082* | 84 904.7 | *-128.05* | 1257.5 | *-98.32* | 4.00 | *0.0291* |
|  | This work | spd functions** | 2.109 | *-0.0182* | 85 994.4 | *-1217.75* | 1525.3 | *-366.12* | 3.92 | *0.1091* |
|  | This work | Full functions* | 2.104 | *-0.0132* | 86 393.90 | *-1617.25* | 1302.9 | *-143.72* | 3.94 | *0.0891* |
| **C^1^П** | **[1]** | **Exp.** | **1.04_9_** |  | **105 820** |  | **2636** |  | **16.0** |  |
|  | This work | spd functions* | 1.038 | *0.002* | 105 211.4 | *608.6* | 2639.4 | *-3.4* | 16.32 | *-0.32* |
|  | This work | spd functions** | 0.987 | *0.053* | 113 844.1 | *-8024.1* | 3145.9 | *-509.9* | 18.05 | *-2.05* |
|  | This work | Full functions* | 1.035 | *0.005* | 107 029.7 | *-1209* | 2925.5 | *-289.5* | 16.40 | *-0.40* |

[1] K. P. Huber and G. Herzberg. Molecular Spectra and Molecular Structure IV. Constants of Diatomic Molecules. Van Nostrand Reinhold Company, New York, 1979. doi: 10.1007/978-1-4757 0961-2.

* Excluding electrons in d-type atomic orbitals.

** Including electrons in d-type atomic orbitals.
