# Supplementary material for: First Principles Rovibronic Absorption Spectra of HF Molecule
Source: J Comput Chem. 2026 Feb 24;47(6):e70317. doi: 10.1002/jcc.70317 (PMC12930379; doi:10.1002/jcc.70317)
Supplement: Supplementary file 1 — Figure S1: The initial and fitted/interpolated transition dipole moment curves for the B–X and C–X transitions. [file JCC-47-0-s001.zip › TS2.docx]

**Table TS2:** Spectroscopic constants of *ab initio* Λ−S electronic states of the HF molecule. Values that include subscripts indicate the last digit's uncertainty, as described by Huber and Herzberg [39]; actual uncertainties may exceed $\pm$10 units of the last digit. Numbers in parentheses (Obs. − Calc.) are based on the only available experimental data [39], given in italics.

| **States** | **Method** | **Ref.** | **R_e_**  **(Å)** | **T_e_**  **(cm^-1^)** | **ω_e_**  **(cm^-1^)** | **ω_e_*x*_e_**  **(cm^-1^)** | **B_e_**  **(cm^-1^)** | **D_e_**  **(eV)** | **μ_e_**  **(ea_0_)** |
| --- | --- | --- | --- | --- | --- | --- | --- | --- | --- |
| **X ^1^Σ^+^** | Expt. | [39] | 0.91680_8_ |  | 4138.32 | 89.88 | 20.9557 | 6.12 |  |
|  | MRCI+Q | This work | 0.917 (-0.0002) | 0.0 | 4098.3 (40.02) | 79.36 | 20.96 (-0.0043) | 5.92 (0.2) | 0.698 |
|  | DPF | [52] | 0.91683897(-0.00003897) |  |  |  |  | 6.12 (0.0) |  |
|  | SCF | [27] | 0.899 (0.0178) |  | 4536 (-397.68) |  | 21.8 (-0.8443) | 4.28 (1.84) |  |
|  | CI | [27] | 0.920 (-0.0032) |  | 4210 (-71.68) |  | 20.8 (0.1557) | 5.88 (0.24) |  |
|  | IVO-CASCI | [28] | 0.926 (-0.0092) |  | 4141.0 (-2.68) |  |  | 5.64 (0.48) | 0.68 |
|  | HV3rd | 28] | 0.910 (0.0068) |  | 4184.9 (-46.58) |  |  | 5.13 (0.99) |  |
|  | MRDCI | [29] | 0.923 (-0.0062) |  | 4148.6 (-10.28) |  | 20.32 (0.6357) | 5.98 (0.14) |  |
|  | MCSCF | [30] | 0.91 (0.0068) |  | 4128.3 (10.02) |  |  | 5.77 (0.35) |  |
|  | CEPA | [31] | 0.916 (0.0008) |  | 4169.3 (-30.98) |  |  | 5.83 (0.29) |  |
|  | PNO-CI | [31] | 0.911 (0.0058) |  | 4251.5 (-113.18) |  |  | 5.69 (0.43) |  |
|  | GVB-CI | [32] | 0.919 (-0.0022) |  | 4158.0 (-19.68) |  |  | 5.72 (0.4) |  |
|  | iCAS-CI | [33] | 0.922 (-0.0052) |  | 4095.0 (43.32) |  | 20.73 (0.2257) | 6.02 (0.1) |  |
|  | DMBPT | [34] | 0.905 (0.0118) |  | 4329.0 (-190.68) |  | 21.53 (-0.5743) |  |  |
|  | MCSCF | [35] | 0.919 (-0.0022) |  | 4102.0 (36.32) |  | 20.84 (0.1157) |  |  |
|  | MRCI | [36] | 0.917 (-0.0002) |  | 4122(16.32) |  | 20.95 (0.0057) | 6.12 (0.0) |  |
|  | MRCI+Q | [37] | 0.916 (0.0008) |  | 4124 (14.32) | 78.72 | 20.99 (-0.0343) | 6.07 (0.05) |  |
|  | i-DMFT | [38] | 0.908 (0.0088) |  | 4283.5 (-145.18) | 90.45 | 21.37 (-0.4143) | 6.14 (-0.02) |  |
| **(2) ^3^Π** | MRCI+Q | This work | 1.038 | 103 890.5 | 2868.61 | 34.03 | 16.36 | 3.254 | 1.81 |
|  | MRDCI | [29] |  | 102 593 |  |  |  |  |  |
|  | iCAS-CI | [33] | 1.032 | 102 432 | 2864 |  | 16.6 |  |  |
|  | MRCI | [36] | 1.006 | 108 563 | 4672.5 | 245.39 |  | 17.59 |  |
|  | MRCI+Q | [37] | 1.028 | 105 520 | 2892 | 74.14 | 16.62 | 3.278 |  |
| **(2) ^3^Σ^+^** | MRCI+Q | This work | 1.003 | 106 382.8 | 4272.4 | 594.011 | 17.54 | 2.916 | 0.28 |
|  | MRCI | [36] | 0.998 | 107 657 | 3492 |  | 17.68 |  |  |
|  | MRCI+Q | [37] | 1.006 | 105 834 | 3450 | 117.26 | 16.93 | 3.214 |  |
| **(1) ^3^Δ** | MRCI+Q | This work | 1.003 | 108 059.9 | 3055.89 |  | 17.48 | 3.077 | 0.27 |
|  | MRDCI | [29] |  | 103 964 |  |  |  |  |  |
|  | iCAS-CI | [33] | 1.006 | 104 851 | 3087 |  | 17.4 |  |  |
|  | MRCI | [36] | 0.997 | 109 281 | 3108 |  | 17.72 |  |  |
|  | MRCI+Q | [37] | 1.005 | 106 869 | 3051 | 89.05 | 17.48 | 3.090 |  |
| **(1) ^3^Σ^-^** | MRCI+Q | This work | 1.004 | 109 382.9 | 3035.8 | 82.54 | 17.43 | 3.008 | 0.27 |
|  | MRCI | [36] | 0.996 | 110481 | 3108 |  | 17.76 |  |  |
|  | MRCI+Q | [37] | 1.004 | 107 724 | 3051 | 91.34 | 17.45 | 2.996 |  |
| **(1) ^1^Δ** | MRCI+Q | This work | 0.997 | 109 584.9 | 3050.6 | 93.29 | 17.73 | 2.98 | 0.23 |
|  | MRDCI | [29] |  |  | 105 012.5 |  |  |  |  |
|  | iCAS-CI | 331 | 1.006 | 104 044.7 | 3078 |  | 17.4 |  |  |
|  | MRCI | [36] | 0.997 | 109 603.2 | 3100 |  | 17.72 |  |  |
|  | MRCI+Q | [37] | 1.007 | 107 454 | 3050 | 91.66 | 17.35 | 3.006 |  |
| **(1) ^1^Σ^-^** | MRCI+Q | This work | 0.997 | 110 070.4 | 3050.7 | 93.18 | 17.71 | 2.923 | 0.23 |
|  | MRCI+Q | (37) | 1.008 | 107 724 | 3053 | 92.19 | 17.28 | 2.970 |  |
| **(3) ^3^Π** | MRCI+Q | This work | 1.034 | 111 118.7 | 3120.5 | 83.32 | 16.40 | 2.724 | 1.57 |
|  | MRCI | [36] | 1.196 | 135 721 | 1524 |  | 12.99 |  |  |
|  | MRCI+Q | [37] | 1.047 | 111 386 | 2846 | 81.17 | 16.08 | 2.530 |  |
| **(3) ^1^Π** | MRCI+Q | This work | 1.039 | 112 116.6 | 3059.3 | 87.44 | 16.33 | 2.604 | 1.228 |
|  | MRCI+Q | [37] | 1.052 | 111 692 | 2877 | 82.16 | 15.91 | 2.487 |  |
| **(3) ^1^Σ^+^** | MRCI+Q | This work | 1.044 | 117 859.7 | 3056.5 | 190.95 | 16.17 | 1.92 | 0.04 |
|  | MRDCI | [29] |  | 113 241.17 |  |  |  |  |  |
|  | iCAS-CI | [33] | 1.048 | 104 852.94 | 3557.0 |  | 16.0 |  |  |
|  | MRCI+Q | [37] | 1.103 | 112 997 | 3099 |  | 14.46 | 2.315 |  |
| **(3) ^3^Σ^+^** | MRCI+Q | This work | 1.009 | 126 111.5 | 3110.07 | 106.01 | 17.37 |  | 0.90 |
|  | MRCI+Q | [37] | 1.003 | 117 912 | 3351 |  | 17.52 | 1.756 |  |
| **(4) ^1^Σ^+^** | MRCI+Q | This work | 1.006 | 126 791.6 | 3262.78 | 161.38 | 17.42 | 0.86 | 0.66 |
|  | MRDCI | [29] |  | 114 531.67 |  |  |  |  |  |
|  | iCAS-CI | [33] | 1.102 | 104 852.94 | 3276.9 |  | 14.5 |  |  |
|  | MRCI+Q | [37] | 1.006 | 117 706 | 3685 |  | 17.23 | 1.714 |  |
