# Supplementary material for: First Principles Rovibronic Absorption Spectra of HF Molecule
Source: J Comput Chem. 2026 Feb 24;47(6):e70317. doi: 10.1002/jcc.70317 (PMC12930379; doi:10.1002/jcc.70317)
Supplement: Supplementary file 1 — Figure S1: The initial and fitted/interpolated transition dipole moment curves for the B–X and C–X transitions. [file JCC-47-0-s001.zip › TS3.docx]

Table TS3: The ro-vibrational constants of the ab initio X ^1^Σ^+^, B ^1^Σ^+^, and C ^1^Π states of the HF molecule. The values in parentheses represent the experimental uncertainties in the last digit(s).

| **State** | **Ref.** | ***v*** | **E*_v_***  **(cm^-1^)** | **B*_v_***  **(cm^-1^)** | **D*_v_*** $\boldsymbol{\times}\boldsymbol{10}^{\boldsymbol{3}}$  **(cm^-1^)** | **R_min_**  **(Å)** | **R_max_**  **(Å)** |
| --- | --- | --- | --- | --- | --- | --- | --- |
| **X ^1^Σ^+^** | This work | 0 | 0 | 20.56 | 2.18 | 0.833 | 1.020 |
|  | [17] |  | 0 | 20.5596(2) | 2.117(2) |  |  |
|  | [58] |  |  | 20.5597300(25) | 2.11991(14) |  |  |
|  | [59] |  |  | 20.5597300(3) | 2.11987(1) |  |  |
|  | This work | 1 | 3 909.34 | 19.77 | 2.11 | 0.783 | 1.114 |
|  | [17] |  | 3 961.418(3) | 19.7872(4) | 2.059(3) |  |  |
|  | This work | 2 | 7 648.58 | 19.01 | 2.05 | 0.754 | 1.188 |
|  | [17] |  | 7 750.814(6) | 19.0328(10) | 1.98(5) |  |  |
|  | This work | 3 | 11 222.38 | 18.26 | 1.99 | 0.732 | 1.256 |
|  | [16] |  | 11 372.807(7) | 18.2995(5) | 1.940(3) |  |  |
|  | This work | 4 | 14 634.12 | 17.53 | 1.94 | 0.715 | 1.321 |
|  | [16] |  | 14 831.622(7) | 17.5829(7) | 1.908(7) |  |  |
|  | This work | 5 | 17 886.73 | 16.82 | 1.90 | 0.701 | 1.384 |
|  | [16] |  | 18 130.966(10) | 16.8792(8) | 1.864(10) |  |  |
|  | This work | 6 | 20 982.47 |  |  | 0.689 | 1.447 |
|  | [15] |  | 21 273.69(10) |  |  |  |  |
|  | This work | 7 | 23 922.9 |  |  | 0.679 | 1.511 |
|  | [15] |  | 24 262.18(6) |  |  |  |  |
|  | This work | 8 | 26 708.69 |  |  | 0.670 | 1.575 |
|  | [15] |  | 27 097.87(4) |  |  |  |  |
|  | This work | 9 | 29 339.66 |  |  | 0.662 | 1.642 |
|  | [15] |  | 29 781.33(5) |  |  |  |  |
|  | This work | 10 | 31 814.42 |  |  | 0.655 | 1.711 |
|  | [19] |  | 32 311.79(7) |  |  |  |  |
|  | This work | 11 | 34 130.02 |  |  | 0.649 | 1.783 |
|  | [19] |  | 34 687.32(7) |  |  |  |  |
| **State** | **Ref.** | ***v*** | **E*_v_***  **(cm^-1^)** | **B*_v_***  **(cm^-1^)** | **D*_v_*** $\boldsymbol{\times}\boldsymbol{10}^{\boldsymbol{4}}$  **(cm^-1^)** | **R_min_**  **(Å)** | **R_max_**  **(Å)** |
| **B ^1^Σ^+^** | This work | 0 | 609.16 | 3.98 | 1.87 | 1.938 | 2.280 |
|  | [19] |  | 572.39 | 4.0200(3) | 2.020(8) |  |  |
|  | This work | 1 | 1767.72 | 3.97 | 2.23 | 1.822 | 2.432 |
|  | [19] |  | 1696.20 | 4.0005(4) | 2.220(9) |  |  |
|  | This work | 2 | 2869.24 | 3.95 | 2.50 | 1.744 | 2.546 |
|  | [19] |  | 2785.60 | 3.9796(5) | 2.425(13) |  |  |
|  | This work | 3 | 3927.41 | 3.93 | 2.68 | 1.684 | 2.647 |
|  | [19] |  | 3841.72 | 3.9581(5) | 2.641(13) |  |  |
|  | This work | 4 | 4949.06 | 3.90 | 2.83 | 1.633 | 2.741 |
|  | [19] |  | 4865.76 | 3.9355(6) | 2.857(15) |  |  |
|  | This work | 5 | 5940.08 | 3.88 | 3.31 | 1.588 | 2.829 |
|  | [19] |  | 5858.84 | 3.9125(6) | 3.097(16) |  |  |
|  | This work | 6 | 6894.69 | 3.85 | 3.42 | 1.547 | 2.918 |
|  | [19] |  | 6821.82 | 3.8902(6) | 3.354(22) |  |  |
|  | This work | 7 | 7819.07 | 3.82 | 3.80 | 1.510 | 3.003 |
|  | [19] |  | 7755.95 | 3.8682(6) | 3.720(28) |  |  |
|  | This work | 8 | 8711.64 | 3.79 | 4.02 | 1.476 | 3.087 |
|  | [19] |  | 8662.27 | 3.8434(6) | 3.944(42) |  |  |
|  | This work | 9 | 9577.31 | 3.77 | 4.35 | 1.443 | 3.170 |
|  | [19] |  | 9541.58 | 3.8197(7) | 4.238(37) |  |  |
|  | This work | 10 | 10416.64 | 3.75 | 4.90 | 1.413 | 3.253 |
|  | [19] |  | 10394.87 | 3.7962(20) | 4.56(12) |  |  |
|  | This work | 11 | 11226.90 | 3.72 | 4.83 | 1.384 | 3.336 |
|  | This work | 12 | 12015.27 | 3.69 | 5.63 | 1.356 | 3.419 |
|  | This work | 13 | 12777.06 |  |  | 1.330 | 3.502 |
|  | This work | 14 | 13515.63 |  |  | 1.305 | 3.585 |
|  | [19] |  | 13566.43 |  |  |  |  |
|  | This work | 15 | 14232.12 |  |  | 1.281 | 3.669 |
|  | [19] |  | 14302.82 |  |  |  |  |
|  | This work | 16 | 14927.75 |  |  | 1.258 | 3.754 |
|  | [19] |  | 15018.24 |  |  |  |  |
|  | This work | 17 | 15600.76 |  |  | 1.236 | 3.840 |
|  | [19] |  | 15713.20 |  |  |  |  |
|  | This work | 18 | 16255.18 |  |  | 1.214 | 3.926 |
|  | [19] |  | 16388.65 |  |  |  |  |
|  | This work | 19 | 16889.14 |  |  | 1.194 | 4.014 |
|  | [19] |  | 17044.95 |  |  |  |  |
|  | This work | 20 | 17506.01 |  |  | 1.174 | 4.102 |
|  | [19] |  | 17682.77 |  |  |  |  |
|  | This work | 21 | 18103.59 |  |  | 1.156 | 4.192 |
|  | [19] |  | 18302.66 |  |  |  |  |
|  | This work | 22 | 18683.45 |  |  | 1.138 | 4.283 |
|  | [19] |  | 18905.03 |  |  |  |  |
| **State** | **Ref.** | ***v*** | **E*_v_***  **(cm^-1^)** | **B*_v_***  **(cm^-1^)** | **D*_v_*** $\boldsymbol{\times}\boldsymbol{10}^{\boldsymbol{3}}$  **(cm^-1^)** | **R_min_**  **(Å)** | **R_max_**  **(Å)** |
| C ^1^Π | This work | 0 | 1334.07 | 16.03 | 2.35 | 0.935 | 1.167 |
|  | [20] |  |  | 16.033 | -0.7 |  |  |
|  | [23] |  |  | 16.03 ± 0.01 | -0.65 ± 0.42 |  |  |
|  | This work | 1 | 3920.87 | 15.38 | 2.22 | 0.875 | 1.281 |
|  | [20] |  |  | 15.473 | 0.9 |  |  |
|  | This work | 2 | 6397.17 | 14.69 | 2.13 | 0.840 | 1.375 |
|  | [20] |  |  | 15.009 | 1.8 |  |  |
|  | This work | 3 | 8748.50 | 13.98 | 2.07 | 0.815 | 1.462 |
|  | [20] |  |  | 14.61 |  |  |  |
|  | This work | 4 | 10966.84 | 13.26 | 2.03 | 0.796 | 1.547 |
