# Supplementary material for: First Principles Rovibronic Absorption Spectra of HF Molecule
Source: J Comput Chem. 2026 Feb 24;47(6):e70317. doi: 10.1002/jcc.70317 (PMC12930379; doi:10.1002/jcc.70317)
Supplement: Supplementary file 1 — Figure S1: The initial and fitted/interpolated transition dipole moment curves for the B–X and C–X transitions. [file JCC-47-0-s001.zip › TS4.docx]

**Table TS4:** Values of the vibrational energy $E_{v}$ , the rotational constant $B_{v}$, the centrifugal constant $D_{v}$ and the abscissas of the turning points R_min_ and R_max_ for the different vibrational levels of excited singlet and triplet states (1)^1^∆, (3)^1^Π, (1)^3^∆, (2)^3^Π, (3)^3^Π, and (1)^3^Σ^-^ of the HF molecule.

| **State** | $\boldsymbol{v}$ | $\boldsymbol{E}_{\boldsymbol{v}}$  **(cm^-1^)** | $\boldsymbol{B}_{\boldsymbol{v}}$  **(cm^-1^)** | $\boldsymbol{D}_{\boldsymbol{v}}$**×10^3^**  **(cm^-1^)** | **R_min_**  **(Å)** | **R_max_**  **(Å)** |
| --- | --- | --- | --- | --- | --- | --- |
| **(1)^1^Δ** | 0 | 1508.82 | 17.26 | 2.35 | 0.902 | 1.120 |
|  | 1 | 4374.82 | 16.33 | 2.30 | 0.848 | 1.235 |
|  | 2 | 7047.04 | 15.43 | 2.26 | 0.817 | 1.332 |
|  | 3 | 9529.14 | 14.53 | 2.23 | 0.794 | 1.423 |
| **State** | $\boldsymbol{v}$ | $\boldsymbol{E}_{\boldsymbol{v}}$  **(cm^-1^)** | $\boldsymbol{B}_{\boldsymbol{v}}$  **(cm^-1^)** | $\boldsymbol{D}_{\boldsymbol{v}}$**×10^3^**  **(cm^-1^)** | **R_min_**  **(Å)** | **R_max_**  **(Å)** |
| **(3)^1^Π** | 0 | 1506.86 | 16.02 | 1.89 | 0.942 | 1.159 |
|  | 1 | 4389.56 | 15.36 | 1.93 | 0.883 | 1.270 |
|  | 2 | 7097.23 | 14.70 | 1.93 | 0.849 | 1.360 |
|  | 3 | 9642.04 | 14.04 | 1.91 | 0.823 | 1.445 |
| **State** | $\boldsymbol{v}$ | $\boldsymbol{E}_{\boldsymbol{v}}$  **(cm^-1^)** | $\boldsymbol{B}_{\boldsymbol{v}}$  **(cm^-1^)** | $\boldsymbol{D}_{\boldsymbol{v}}$**×10^3^**  **(cm^-1^)** | **R_min_**  **(Å)** | **R_max_**  **(Å)** |
| **(1)^3^Δ** | 0 | 1555.71 | 17.23 | 2.20 | 0.905 | 1.120 |
|  | 1 | 4530.75 | 16.46 | 2.30 | 0.849 | 1.231 |
|  | 2 | 7259.35 | 15.42 | 2.23 | 0.817 | 1.327 |
| **State** | $\boldsymbol{v}$ | $\boldsymbol{E}_{\boldsymbol{v}}$  **(cm^-1^)** | $\boldsymbol{B}_{\boldsymbol{v}}$  **(cm^-1^)** | $\boldsymbol{D}_{\boldsymbol{v}}$**×10^3^**  **(cm^-1^)** | **R_min_**  **(Å)** | **R_max_**  **(Å)** |
| **(2)^3^Π** | 0 | 1419.45 | 16.10 | 2.15 | 0.936 | 1.157 |
|  | 1 | 4144.35 | 15.45 | 2.25 | 0.875 | 1.275 |
|  | 2 | 6679.74 | 14.71 | 2.04 | 0.841 | 1.368 |
| **State** | $\boldsymbol{v}$ | $\boldsymbol{E}_{\boldsymbol{v}}$  **(cm^-1^)** | $\boldsymbol{B}_{\boldsymbol{v}}$  **(cm^-1^)** | $\boldsymbol{D}_{\boldsymbol{v}}$**×10^3^**  **(cm^-1^)** | **R_min_**  **(Å)** | **R_max_**  **(Å)** |
| **(3)^3^Π** | 0 | 1537.38 | 16.18 | 1.88 | 0.905 | 1.119 |
|  | 1 | 4457.65 | 15.44 | 2.01 | 0.850 | 1.228 |
|  | 2 | 7150.99 | 14.72 | 1.89 | 0.818 | 1.324 |
| **State** | $\boldsymbol{v}$ | $\boldsymbol{E}_{\boldsymbol{v}}$  **(cm^-1^)** | $\boldsymbol{B}_{\boldsymbol{v}}$  **(cm^-1^)** | $\boldsymbol{D}_{\boldsymbol{v}}$**×10^3^**  **(cm^-1^)** | **R_min_**  **(Å)** | **R_max_**  **(Å)** |
| **(1)^1^Σ^-^** | 0 | 1569.96 | 17.25 | 2.18 | 0.066 | 1.117 |
|  | 1 | 4553.16 | 16.44 | 2.38 | 0.066 | 1.232 |
|  | 2 | 7252.10 | 15.31 | 2.17 | 0.066 | 1.329 |
