# Supplementary figures and images for: First Principles Rovibronic Absorption Spectra of HF Molecule
Source: J Comput Chem. 2026 Feb 24;47(6):e70317. doi: 10.1002/jcc.70317 (PMC12930379; doi:10.1002/jcc.70317)

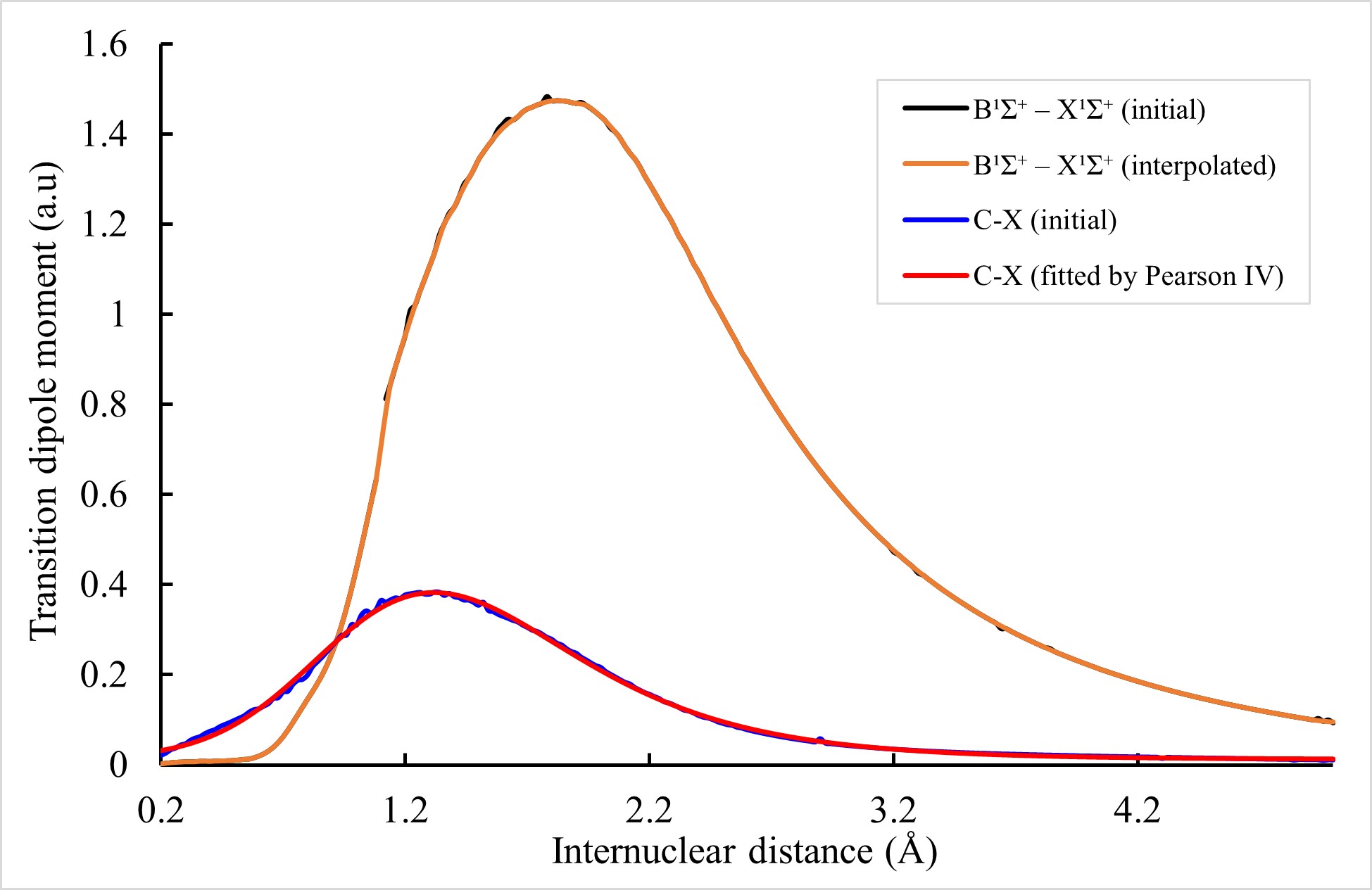

Supplement: Supplementary file 1 — Figure S1: The initial and fitted/interpolated transition dipole moment curves for the B–X and C–X transitions. [file JCC-47-0-s001.zip › Fig. SI.jpg]
